# Network‐based cytokine inference implicates Oncostatin M as a driver of an inflammation phenotype in knee osteoarthritis

**DOI:** 10.1111/acel.14043

**Published:** 2023-12-18

**Authors:** Hirotaka Iijima, Fan Zhang, Fabrisia Ambrosio, Yusuke Matsui

**Affiliations:** ^1^ Discovery Center for Musculoskeletal Recovery Schoen Adams Research Institute at Spaulding Charlestown Massachusetts USA; ^2^ Department of Physical Medicine & Rehabilitation Harvard Medical School Boston Massachusetts USA; ^3^ Department of Physical Medicine & Rehabilitation Spaulding Rehabilitation Hospital Charlestown Massachusetts USA; ^4^ Institute for Advanced Research Nagoya University Nagoya Japan; ^5^ Biomedical and Health Informatics Unit, Graduate School of Medicine Nagoya University Nagoya Japan; ^6^ Department of Medicine Division of Rheumatology University of Colorado School of Medicine Aurora Colorado USA; ^7^ Department of Biomedical Informatics Center for Health AI University of Colorado School of Medicine Aurora Colorado USA; ^8^ Institute for Glyco‐core Research, Tokai National Higher Education and Research System Nagoya University Nagoya Japan

**Keywords:** aging, cartilage‐specific network, cytokine inference, matrix remodeling, network propagation, Oncostatin M, osteoarthritis

## Abstract

Inflammatory cytokines released by synovium after trauma disturb the gene regulatory network and have been implicated in the pathophysiology of osteoarthritis. A mechanistic understanding of how aging perturbs this process can help identify novel interventions. Here, we introduced network paradigms to simulate cytokine‐mediated pathological communication between the synovium and cartilage. Cartilage‐specific network analysis of injured young and aged murine knees revealed aberrant matrix remodeling as a transcriptomic response unique to aged knees displaying accelerated cartilage degradation. Next, network‐based cytokine inference with pharmacological manipulation uncovered IL6 family member, Oncostatin M (OSM), as a driver of the aberrant matrix remodeling. By implementing a phenotypic drug discovery approach, we identified that the activation of OSM recapitulated an “inflammatory” phenotype of knee osteoarthritis and highlighted high‐value targets for drug development and repurposing. These findings offer translational opportunities targeting the inflammation‐driven osteoarthritis phenotype.

## Graphical abstract

The network medicine paradigms introduced here simulated cytokine‐mediated pathological communication between the synovium and cartilage and uncovered IL6 family member, OSM, as a driver of aberrant matrix remodeling. Intriguingly, while OSM is an IL6 family cytokine, pharmacological manipulations suggested that the age‐related aberrant ECM remodeling is attributed, at least partly, to downstream signals of OSM, but not IL6. By implementing a phenotypic drug discovery approach, we identified that the activation of OSM recapitulated an “inflammatory” phenotype of KOA and highlighted high‐value targets for drug development and repurposing. ECM, extracellular matrix; KOA, knee osteoarthritis; OSM, Oncostatin M; OSMR, Oncostatin M receptor.

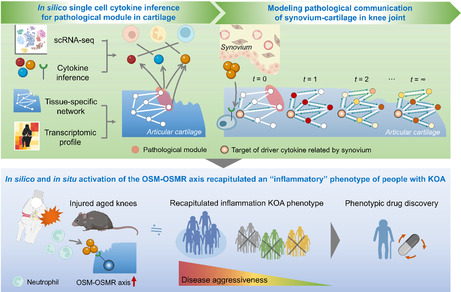

**Abbreviations**
ACL: anterior cruciate ligament
DMM: destabilized medial meniscus
ECM: extracellular matrix
FC: fold change
FDR: false discovery rate
GAG: glycosaminoglycan
GO: gene ontology
KOA: knee osteoarthritis
NES: normalized enrichment score
OARSI score: Osteoarthritis Research Society International score
OSM: oncostatin M
OSMR: oncostatin M receptor
PC: principal component
PCA: principal component analysis
RWR: random‐walk‐restart
SMD: standardized mean difference
ssGSEA: single sample gene set enrichment analysis
TOM: topological overlap matrix
WGCNA: weighted gene correlation network analysis

## 1 INTRODUCTION

Adaptation, or “adaptive homeostasis,” is a highly conserved process, wherein cells, tissues, and whole organisms transiently activate a suite of signaling pathways in response to short‐term external perturbations, thereby effecting transient changes in gene expression and stress resistance (López‐Otín et al., 2013). Mounting evidence suggests, however, that the capacity for adaptive homeostasis declines with aging. In 2014, Kennedy et al suggested seven pillars of aging, one of which was “adaptation to stress,” whereby reduced stress capacity over time is a major risk factor for most late‐onset human diseases (Kennedy et al., 2014). Given the modifiable nature of stress, leveraging the relationship between stress and tissue health represents a promising area for the development of therapeutic interventions to treat aging diseases.

One of the most debilitating age‐related diseases is osteoarthritis (OA) of the knee joint (KOA), which is characterized by an age‐ and/or mechanical stress‐dependent progressive loss of cartilage integrity (Guilak, 2011; Iijima et al., 2022; Loeser et al., 2016). Age‐associated cellular changes in articular cartilage include cell depletion due to various forms of cell death together with dysfunctional intrinsic cellular repair responses to mechanical loading (Lotz & Loeser, 2012). This impaired intrinsic cellular repair response with aging amplifies the deleterious effects of traumatic injury (Houtman et al., 2021; Loeser, Olex, et al., 2012; Madej et al., 2016), for example, through increased levels of extracellular matrix (ECM) degrading enzymes including matrix metalloproteinases (MMPs) (Kurz et al., 2001) and decreased cartilage ECM production (Kurz et al., 2001; Madej et al., 2016). While these age‐related stress responses after trauma are known accelerants of KOA, most studies investigating the transcriptomic response in vivo have utilized relatively young animals (~6 months). Moreover, deleterious mechanical loading through disruption of knee structures and tissues is more common in elderly individuals than has been previously appreciated (Englund et al., 2008; Hasegawa et al., 2012). As a field, we therefore lack both a holistic understanding of the complex KOA disease process as well as age‐dependent disease drivers that may serve as promising therapeutic targets in a geriatric population.

While KOA has historically been considered a disease of “wear and tear,” it is increasingly viewed to be a whole‐joint disease that results from complex tissue–tissue interactions (Loeser, Goldring, et al., 2012), and particularly, cross talk between the synovium and cartilage (Chou et al., 2020). Inflammatory immune mediators have been identified in the context of inflamed synovium from rheumatoid arthritis (Zhang et al., 2019), but it is unclear which cytokines released from the synovium interact with cartilage in KOA. One hypothesis is that inflammatory cytokines released by the synovium disturb the complex gene regulatory network in articular cartilage and have been implicated in the pathophysiology of KOA (Kapoor et al., 2011). To unravel these complex interactions, network‐based cytokine inference offers a novel platform with the potential to guide further experimental work designed to uncover disease mechanisms and cytokine therapeutic targets (Barabási et al., 2011). This network medicine approach postulates a “disease module hypothesis,” in which disease‐associated genes or proteins likely share the same topological neighborhood in a network (Barabási et al., 2011). Network propagation, which simultaneously considers all possible paths between given cytokine target genes and the disease‐associate genes, is, therefore, a powerful tool for elucidating cytokine drivers as novel therapeutic targets. The network‐based cytokine inference integrated with network propagation‐based disease driver discovery is further strengthened by considering tissue specificity to understand complex molecular interactions in the manifestation of diseases (Wong et al., 2018).

In this study, we used a series of network medicine approaches to thoroughly characterize age‐related alterations in the transcriptomic responses to traumatic injury in the knee joint. We started by performing a meta‐analysis of the existing literature to summarize the current knowledge of mechanisms underlying the predisposition of aged knees to display accelerated KOA following trauma. Subsequent network‐based cytokine inference integrated with network propagation predicted Oncostatin M (OSM) and OSM receptor (OSMR) as primary cytokine drivers of aberrant ECM remodeling and stiffening with aging. These in silico findings were then cross‐checked through systems biological analysis of archived RNA‐Seq data for pharmacological manipulation of OSM to murine chondrocytes in vitro. The culmination of these analyses suggests that aberrant ECM remodeling induced by the OSMR drives age‐related accelerated KOA after trauma through pathological synovium–cartilage cross talk. To enhance the clinical relevance of our work, these murine findings were compared with the transcriptomic signature of people with KOA. We discovered that activation of OSM–OSMR axis drives an inflammatory phenotype of KOA, the major phenotype that represents 16%–30% of KOA (Dell'Isola et al., 2016). These data suggest that targeting the OSM–OSMR axis may represent a promising new therapeutic target in the treatment of inflammation phenotype of KOA. Finally, we propose novel drug candidates that reduce OSM–OSMR signaling and reverse inflammation, offering translational opportunities for the treatment of KOA in the clinic.

## 2 RESULTS

### 2.1 Aging aggravates trauma‐induced cartilage degeneration

**FIGURE 1:**
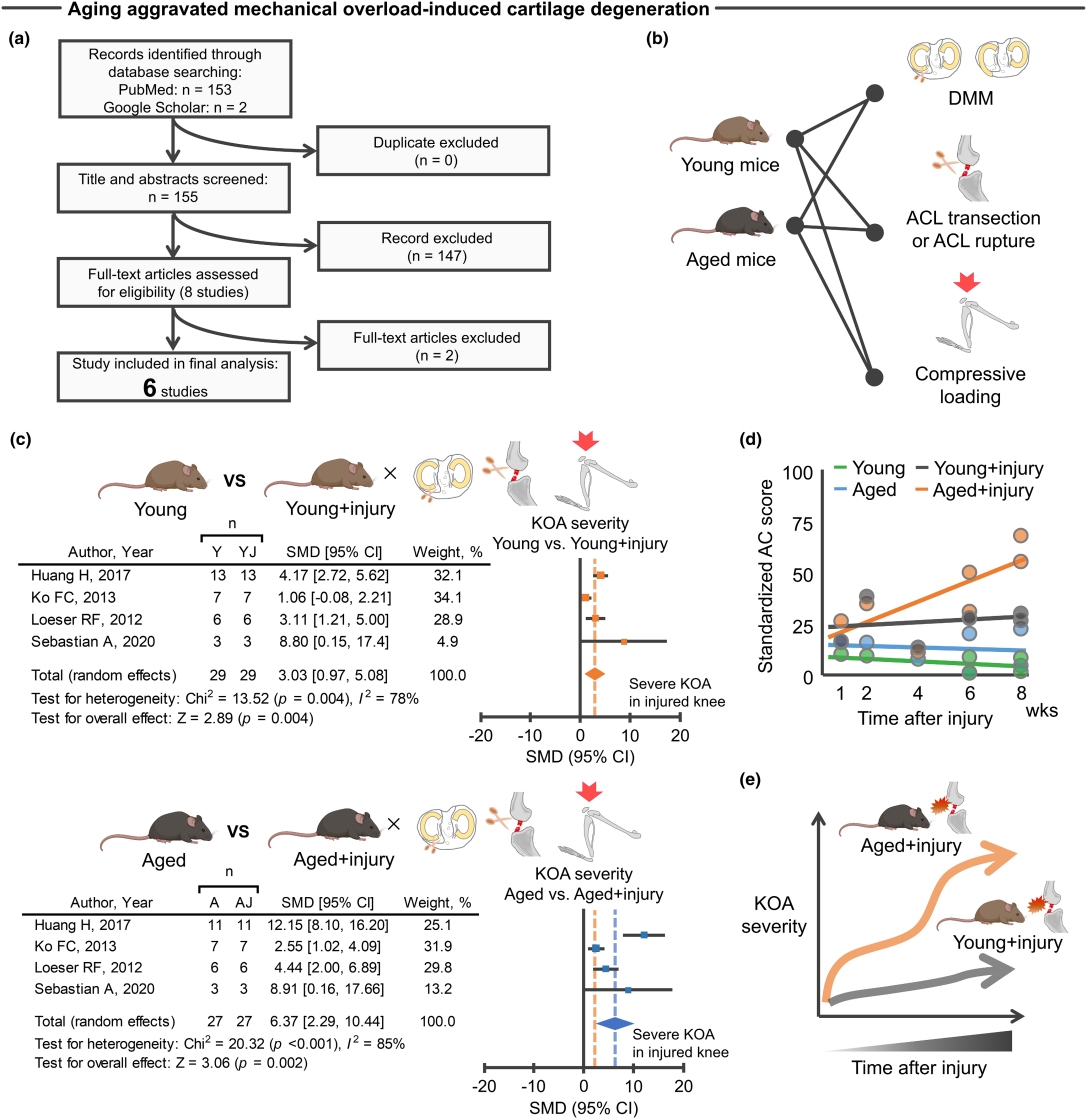
Aged mice displayed accelerated cartilage degeneration after traumatic injury. (a) An electronic database search yielded a total of 155 studies, of which 6 studies were finally included in the meta‐analysis for histological analysis. (b) Mouse models used in the included studies were DMM, ACL transection, ACL rupture, or excessive compressive loading, all of which were used to produce mechanical overloading to knee joint. (c) Meta‐analysis of histological analysis for articular cartilage revealed that mechanical overload induces cartilage degeneration in which aged mice displayed greater cartilage degeneration compared to young mice. The forest plot displays relative weight of the individual studies, SMDs, and 95% CIs. Diamonds indicate the global estimate and its 95% CI. The orange dotted line in the lower panel indicates the average SMD of young mice. (d) A trajectory in cartilage morphology (standardized AC score) across the four different groups (young, aged, young + injury, and aged + injury) revealed that aged, but not young, mice displayed progressive cartilage degeneration after injury. Each dot represents an individual study. (e) Schematic illustrating the trajectory difference in KOA severity between aged and young mice after injury. Portions of the figures were created with Biorender.com. 95% CI, 95% confidence interval; A, aged; AC, articular cartilage; ACL, anterior cruciate ligament; AJ, aged + injury; DMM, destabilized medial meniscus; KOA, knee osteoarthritis; SMD, standardized mean difference; Y, young; YJ, young + injury.

While studies using cartilage explant models have suggested that aging amplifies the deleterious effects of traumatic injury (Houtman et al., 2021; Madej et al., 2016), no comprehensive study to date has summarized age‐related changes in the stress response of trauma to the knee joint in vivo. Therefore, we first sought to address this knowledge gap by performing a systematic review of structural and molecular changes about the knee joint after traumatic injury in both young and aged mice. Six studies met the prespecified inclusion criteria (Faust et al., 2020; Huang et al., 2016, 2017; Ko et al., 2013; Loeser, Goldring, et al., 2012; Loeser, Olex, et al., 2012; Sebastian et al., 2020) (Figure 1a). All identified studies used C57BL/6 background mice (Table S1). The experimental models used included destabilization of the medial meniscus (DMM), anterior cruciate ligament (ACL) transection or rupture, or tibial compressive loading (Figure 1b), all of which produce excessive mechanical loading to the knee joint (Kuyinu et al., 2016).

Subsequent meta‐analysis of histological metrics confirmed that aging provokes a more severe cartilage degeneration after traumatic injury when compared to young counterparts (Figure 1c). For these studies, cartilage degeneration was defined as disruption of cartilage integrity assessed by semiquantitative histological scoring. Notably, aged mice displayed progressive cartilage degeneration after traumatic injury up to 8 weeks after injury, a trend that was not observed in uninjured aged mice, injured young mice, or uninjured young mice (Figure 1d). The trauma‐induced structural changes in the aged knee are supported by non‐pooled data (i.e., data that were not included in the meta‐analysis of histological metrics because of methodological heterogeneity). For example, we found that ACL transection in aged mice resulted in elevated mRNA levels of p16, a biomarker of chondrocyte aging (Diekman et al., 2018), in the knee joint when compared to young counterparts (Faust et al., 2020).

Given that epidemiologic and biologic studies have suggested a link between cartilage degeneration and subchondral bone abnormal remodeling (Iijima et al., 2014, 2016), we also searched for evidence of increased subchondral bone alterations in aged knees after traumatic injury. Meta‐analysis from three studies revealed that excessive mechanical loading to aged knees contributed to increased subchondral bone thickness, a surrogate marker of abnormal bone remodeling (Li et al., 2013), when compared to young counterparts (Figure S1a,b). These studies indicate that the aged joint displays accelerated degenerative changes in the cartilage‐subchondral bone unit after traumatic injury relative to young counterparts (Figure 1e). These data suggest that aging exacerbates trauma‐induced KOA in vivo.

### 2.2 Cartilage‐specific network analysis identified aberrant ECM remodeling as a transcriptomic signature of injured aged knees

As a next step toward a more comprehensive view of the age‐related accelerated KOA after injury, we assessed the global transcriptomic changes over time following a trauma. Only one study to date has performed RNA‐Seq on whole‐joint tissue samples from different age groups over time after trauma (Sebastian et al., 2020). This study, reported by Sebastian et al. (2020), demonstrated that ACL rupture results in significant alterations in genes associated with ECM remodeling and cartilage/bone metabolism in both young and aged mice relative to uninjured controls. These transcript‐level findings are consistent with the disrupted cartilage integrity and increased subchondral bone thickness shown in our meta‐analysis of histological metrics (Figure 1c–e, Figure S1). However, a direct comparison in the transcriptomic response to ACL rupture across the different aged mice was not performed.

**FIGURE 2:**
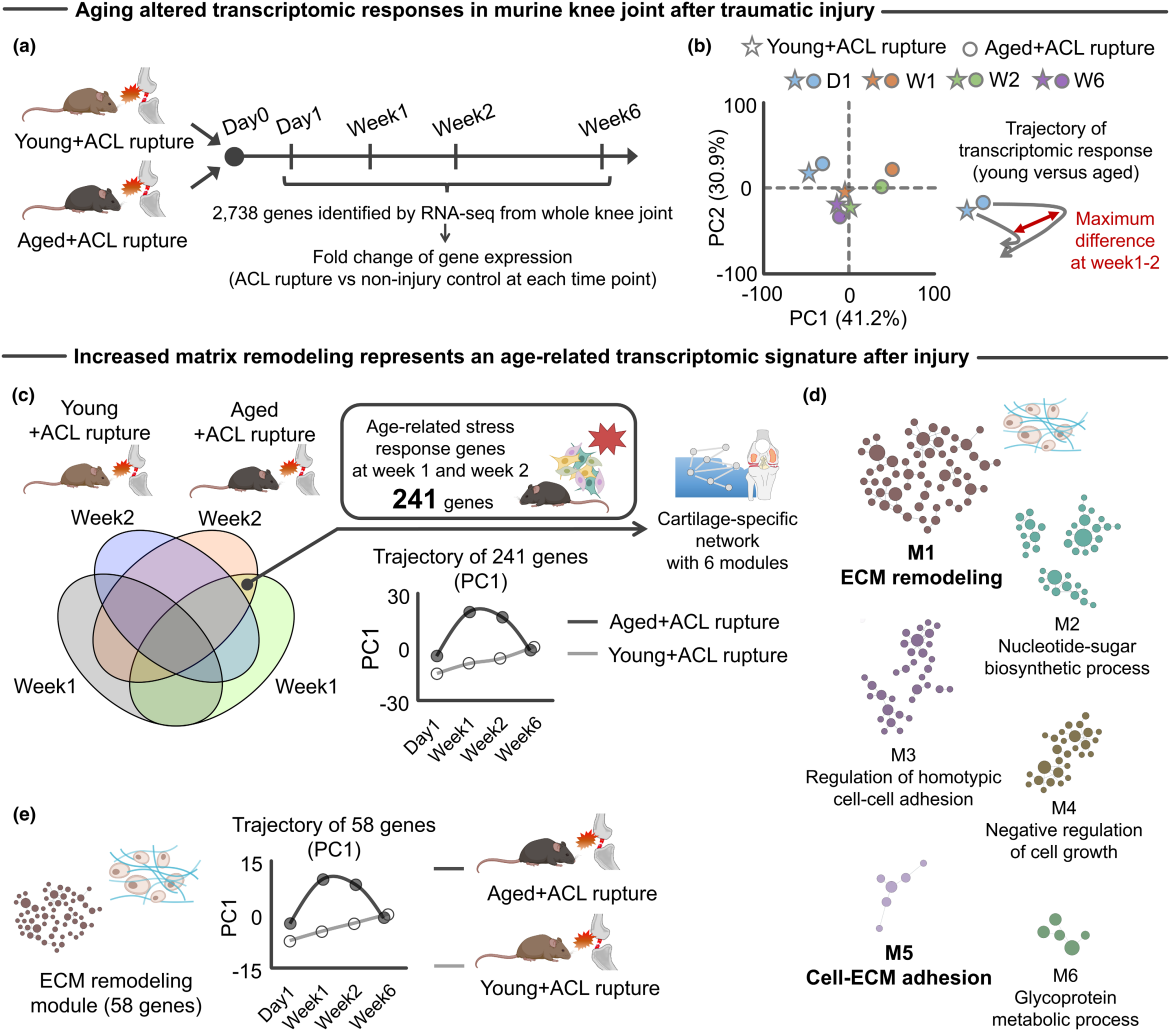
Aberrant ECM remodeling was a transcriptomic signature of aged joint after traumatic injury. (a) Schematic showing the experimental flow of archived RNA‐Seq data (Sebastian et al., 2020). In total, 2738 genes were identified across the different time points (Day1, Week1, Week2, and Week6) after ACL rupture in young (3 months old) and aged (24 months old) C57/BL6 mice (Sebastian et al., 2020). (b) PCA showing the separate trajectory of time course changes (D1, Day1; W1, Week1; W2, Week2; W6, Week6) in the transcript profiles of young and aged mice with the largest difference at Week1 and Week2. (c) We identified 241 age‐related stress response genes that were significantly upregulated in aged + ACL rupture (versus uninjured contralateral knee) across the different time point (Week1 and Week2) but not in young + ACL rupture. PCA revealed PC1 with 241 genes displayed different trajectories between young and aged mice after ACL rupture with the largest difference at Week1 and Week2. From 241 genes, a cartilage‐specific network was constructed using HumanBase software (Greene et al., 2015). (d) The cartilage‐specific network included six modules with the largest module annotated to “ECM remodeling.” Another small module was also annotated to “Cell‐ECM adhesion.” In the cartilage‐specific network, each node and edge represent gene and gene–gene interaction, respectively. (e) PCA revealed that PC1 of ECM remodeling module genes (58 genes) displayed a different trajectory between young and aged mice after ACL rupture, with the largest difference at Week1 and Week2. Portions of the figures were created with Biorender.com. ACL, anterior cruciate ligament; ECM, extracellular matrix; PC, principal component; PCA, principal component analysis.

To better understand age‐related changes in transcriptomic responses underlying the age‐related accelerated KOA, we accessed the archived RNA‐Seq data from young (3 months) and aged (15 months) mice from the study described above (Sebastian et al., 2020). We evaluated expression data of 2738 genes across different time points (Day1, Week1, Week2, and Week6) after ACL rupture (Figure 2a). We then assessed the difference in transcriptomic response (i.e., log_2_ fold change) across the two groups (young + ACL rupture vs. aged + ACL rupture) over time using principal component analysis (PCA). PCA of the time‐course (Day 1 to Week 6) of the transcriptomic response revealed a clear segregation of age groups between Weeks 1 and 2 (Figure 2b). One and 2 weeks after injury represents a phase of active tissue remodeling in response to initiated degenerative cartilage (as shown in Figure 1d). As such, we focused on the 241 overlapping differentially expressed genes (false discovery rate <0.05) that were upregulated at Week1 and Week2 after injury compared to contralateral uninjured counterpart in aged, but not in young, mice (Figure 2c). The complete list of 241 genes is provided in Table S2. We labeled these 241 genes “*age‐related stress response genes*.” Of note, only 14 genes were significantly upregulated at Week1 and Week2 after injury in young, but not aged, mice. As these genes are relatively few in number, this study did not perform subsequent analyses on the young‐related stress response genes.

We next sought to characterize the biological function of the age‐related stress response genes identified. For this purpose, we generated a cartilage‐specific functional gene network using the HumanBases software, which is a genome‐scale protein function and interaction map of human tissues derived from integrating datasets from thousands of experiments (Greene et al., 2015) (Figure 2c). A functional relationship implies that both genes participate in the same biological process in a specific tissue or functional context (Wong et al., 2018). The tissue‐specific functional gene network clarifies biological functions of given genes to help identify disease driver genes that may exert important functional roles according to the tissue of interest (Lage et al., 2008; Reverter et al., 2008; Wong et al., 2018).

In total, six modules were detected from the cartilage‐specific network, with the biggest module (58 genes) representing “ECM remodeling.” Accordingly, “Cell‐ECM adhesion” represented another module that emerged (Figure 2d). Of interest was the ECM remodeling module, which displayed distinct trajectories over time across the two age groups (Figure 2e). ECM remodeling, a dynamic process of synthesis, degradation, and turnover of the complex network of macromolecules that form ECM, is actively involved in the onset and/or development of various diseases, including KOA (Bonnans et al., 2014). The shift in ECM‐related gene expression is in line with a previous study demonstrating that mechanical overloading to elderly osteochondral explants significantly changed the gene ontology (GO) biological process, “focal adhesion” (Houtman et al., 2021). Focal adhesion is a biological function directly triggered by ECM remodeling (Baker et al., 2015). We note that the ECM remodeling module was not identified as the primary biological function when using either traditional GO enrichment analysis (Enrichr software (Kuleshov et al., 2016); Figure S2) or non‐cartilage‐specific (i.e., lung, bone, and heart) network analysis (Greene et al., 2015) (Figure S3). This observation highlights the significance of considering the tissue‐dependent network topology in interpreting biological function of genes.

We also identified 77 genes that were significantly downregulated 1 and 2 weeks after injury compared to contralateral uninjured counterparts in aged, but not young, mice (Figure S4, Table S2). We again characterized the biological function of these 77 genes by implementing a cartilage‐specific functional gene network, which identified only one module representing a “Response to oxidative stress.” These new findings are in line with previous studies showing that aged cartilage displays decreased resistance to oxidative stress (Iijima et al., 2022), potentially leading to aberrant ECM remodeling (Martins et al., 2021). Of note, when we considered all 318 genes (i.e., 241 upregulated + 77 downregulated genes) for the cartilage‐specific functional gene network analysis, we still identified a large module annotated as “ECM remodeling” (Figure S5). Together, we interpreted these results to suggest that ECM remodeling represents a primary biological function uniquely involved in the pathological process of accelerated KOA in aged mice after traumatic injury, processes that may be attributed to reduced resistance to oxidative stress.

Increased ECM remodeling was also identified on the cartilage‐specific network using a second set of transcriptomic data of aged murine knee joint at 8 weeks after DMM induction (Loeser, Olex, et al., 2012) (Figure S6), suggesting overlapping transcriptomic responses associated with ECM remodeling across different trauma models. In addition, we found that 726 genes upregulated after ACL rupture in both young and aged murine knee joints were also significantly associated with ECM remodeling (Figure S7a,b). For all of these comparisons, the aged knee joint displayed a greater transcriptomic response relative to young counterparts (Figure S7c). Among the genes of interest, we identified *Lox* as significantly upregulated by ACL rupture in both young and aged knee joints (Figure S7d). The protein product of *Lox*, lysyl oxidase (LOX), is one of the major enzymes involved in collagen cross‐linking (Kagan & Li, 2003) and has been shown to contribute to pathogenesis of post‐traumatic KOA (Kim et al., 2015). The collective findings from histological and transcriptomic analyses indicate that traumatic injury induces ECM remodeling in murine knee joints, and these transcriptomic responses are further exacerbated by aging.

Given that transcripts were collected from whole‐joint tissue of the knee joint, we also asked whether ECM remodeling after ACL rupture in aged mice is associated with articular chondrocyte markers. As there are no established chondrocyte‐specific markers, we defined a set of articular chondrocyte markers using archived single‐cell RNA‐Seq data from adult murine knee joints (Sebastian et al., 2021). In the single‐cell RNA‐Seq data, 42 genes were predominantly expressed in articular chondrocytes (Table S3). Using this set as a signature, we found that the 241 age‐related stress response genes were significantly enriched in chondrocytes (odds ratio: 5.96; Figure S8). These data suggest that aberrant remodeling of the ECM after injury is primarily driven by aged articular chondrocytes.

### 2.3 Aging amplified the trauma‐induced transcriptomic responses to cartilage ECM stiffening

**FIGURE 3:**
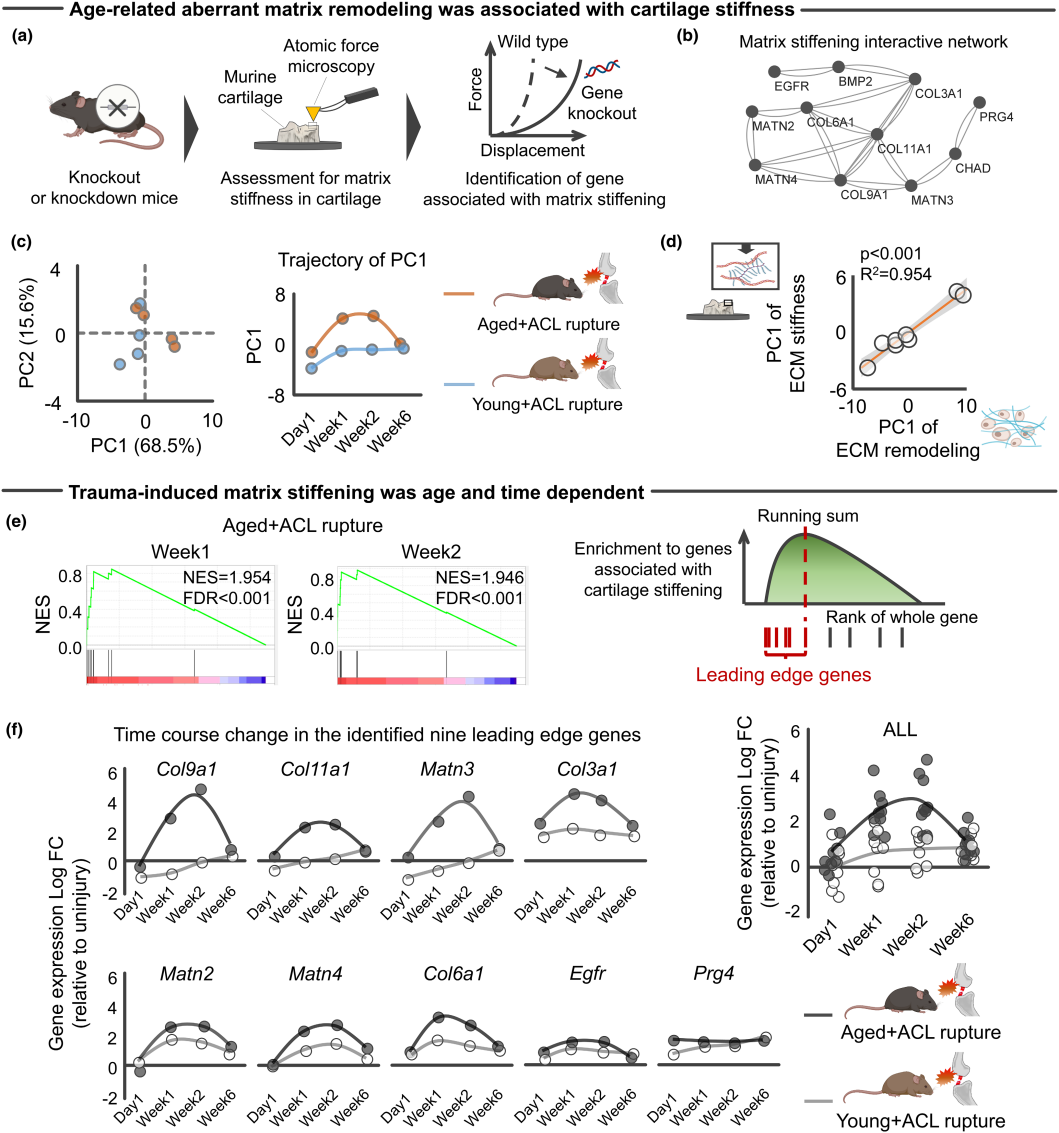
Aging‐specific transcriptomic response to mechanical overload was associated with ECM stiffening in murine knee joint. (a) Schematic showing definition of genes associated with cartilage stiffening. A systematic search identified studies assessing ECM stiffness of murine cartilage with and without gene manipulation using atomic force microscopy. (b) ECM stiffening‐related genes contributed to protein interactive network constructed by String (Szklarczyk et al., 2022). (c) PCA showing the separate trajectory of time course changes in transcripts response of ECM stiffening genes to ACL rupture between young and aged mice. (d) Transcriptomic response of genes related to ECM remodeling was associated with those related to ECM stiffening. (e) ssGSEA revealed that 241 genes upregulated in aged + ACL rupture were significantly enriched to cartilage stiffening. Nine leading‐edge genes were identified by ssGSEA. (f) A time course trajectory of leading‐edge genes stratified by age. Statistical analyses were performed using linear regression (d). Portions of the figures were created with Biorender.com. ACL, anterior cruciate ligament; ECM, extracellular matrix; FC, fold change; FDR, false discovery rate; NES, normalized enrichment score; PC, principal component; PCA, principal component analysis; ssGSEA, single sample gene set enrichment analysis.

ECM remodeling alters biophysical properties of the ECM, often resulting in increased stiffness over time (Stolz et al., 2009). For this reason, matrix alterations have recently been proposed to be a hallmark of aging (Schmauck‐Medina et al., 2022). It is well established that increased ECM stiffness disrupts chondrocyte functionality via mechanotransductive signals (Du et al., 2016; Iijima et al., 2023; Kim et al., 2015; Zhong et al., 2013). We therefore sought to dissect biophysical from biochemical effects of the ECM. Specifically, we tested whether age‐related ECM remodeling genes upregulated after ACL rupture in aged mice are associated with biophysical ECM features. For this purpose, we first defined genes associated with cartilage ECM stiffness in murine knees, as presented in published studies. For this, we again performed a systematic literature search to collect articles that examined cartilage ECM stiffness using atomic force microscopy or similar measurement systems designed to assess biophysical properties of cartilage in mice with and without gene manipulations (Figure 3a). In these analyses, no restriction was applied for the target gene selection. If a genetic modification significantly changed cartilage ECM stiffness, then we defined that gene as associated with cartilage stiffness.

The systematic literature search finally included 19 studies implementing gene knockout/knockdown (Table S4). Of the genes of interest, we finally identified 11 genes (*Bmp2*, *Chad*, *Col3a1*, *Col6a1*, *Col9a1*, *Col11a1*, *Egfr*, *Matn2*, *Matn3*, *Matn4*, and *Prg4*) that were associated with increased cartilage stiffness (i.e., knockout/knockdown significantly decreased cartilage stiffness). To visualize functional relationship among the identified 11 genes, we constructed a protein–protein interactive network via STRINGdb database (Szklarczyk et al., 2022) (Figure 3b). Interestingly, *Col11a1* emerged as a hub gene of the constructed protein–protein interactive network. *Col11a1*, genetically associated with human OA in multiple joints (Boer et al., 2021), is a gene encoding type XI collagen, which normally interacts with Type II and Type IX collagen to form the meshwork of collagen fibrils in articular cartilage (Rodriguez et al., 2004). PCA revealed that the injured aged knee joint displayed different transcriptomic responses of 11 ECM stiffness‐related genes when compared to young counterparts (Figure 3c). As expected, the transcriptomic response of ECM stiffness‐related genes was significantly associated with those of age‐related aberrant ECM remodeling (Figure 3d). We interpret these results to suggest that the aberrant ECM remodeling unique to aged knees is, at least partly, accompanied by ECM stiffening.

To further support the identified relationship between an age‐related altered transcriptomic response to traumatic injury and cartilage stiffening, we performed single‐sample gene set enrichment analysis (ssGSEA) (Barbie et al., 2009) with transcriptomic response of aged knees at Week1 and Week2 after ACL rupture (Sebastian et al., 2020), in which the 11 genes associated with increased ECM stiffness were included as a gene set. An extension of gene set enrichment analysis (GSEA) (Barbie et al., 2009), ssGSEA, calculates separate enrichment scores for each paring of a sample and gene set. The goal of ssGSEA was to prioritize genes significantly correlated with a given phenotype (i.e., ECM stiffening). Using this analysis, we found that ACL rupture in the aged knee increased expression of genes associated with increased cartilage stiffness, in which nine genes (*Col3a1*, *Col6a1*, *Col9a1*, *Col11a1*, *Matn2*, *Mant3*, *Matn4*, *Prg4*, and *Egfr*) were identified as leading edge genes that were maximally upregulated at Week1 and Week2 after ACL rupture in aged, but not young, mice (Figure 3e,f). These results suggest that aging amplifies trauma‐induced ECM stiffening.

### 2.4 Network‐based cytokine inference predicted the OSM–OSMR axis as a driver of age‐related aberrant ECM remodeling

**FIGURE 4:**
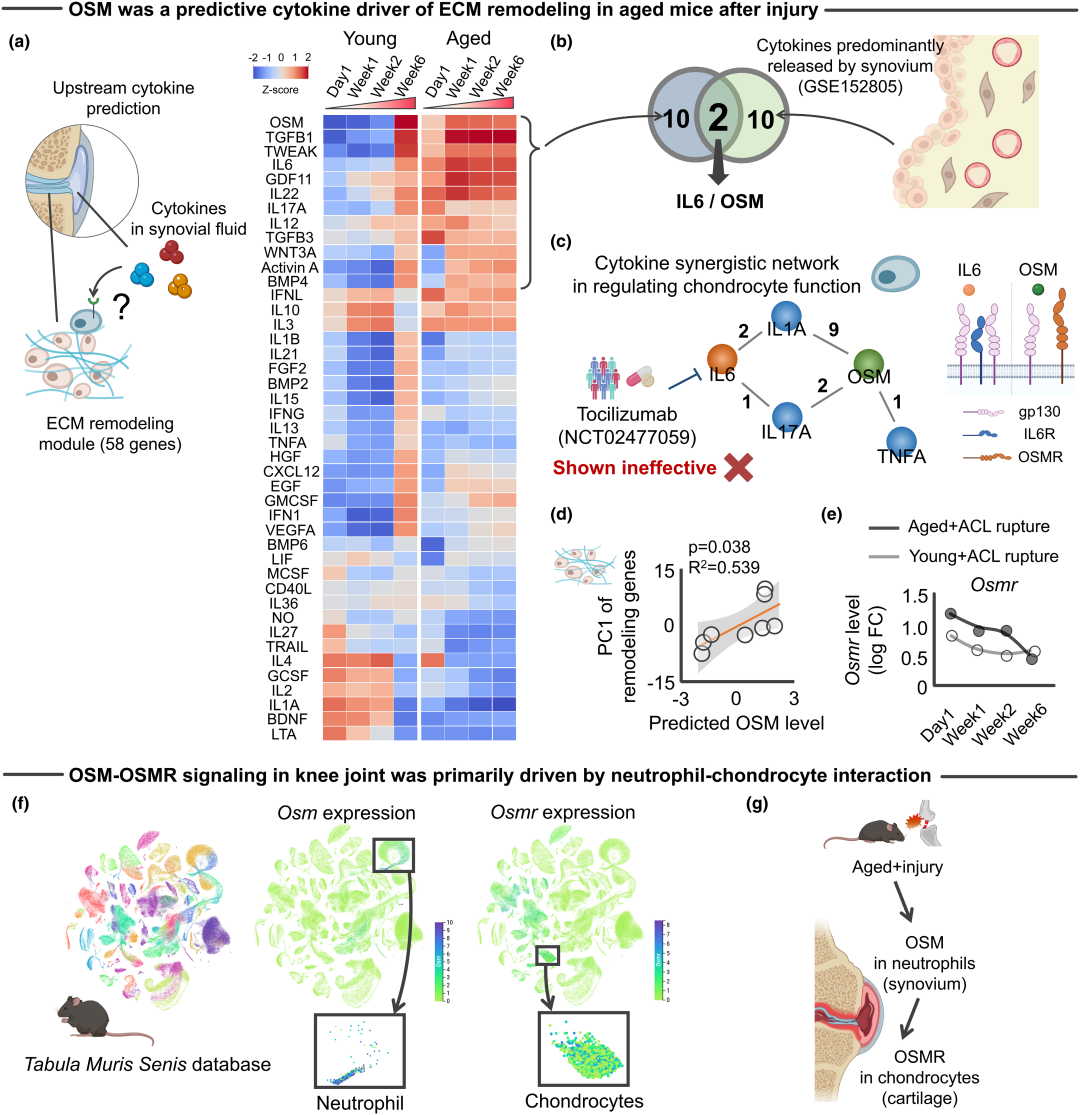
Activated OSM‐OSMR was predicted driver of age‐related aberrant ECM remodeling. (a) Predicted upstream cytokines driving the age‐related aberrant ECM remodeling in the cartilage‐specific network analyzed by CytoSig software (Jiang et al., 2021). Heatmap color indicates *z*‐score for each gene. Of 43 cytokines, 12 were predominantly upregulated in aged knee joints. (b) Integrated analysis of upstream cytokine inference and archived scRNA‐Seq of synovium (GSE152805) (Chou et al., 2020) identified IL6 and OSM as possible cytokine drivers predominantly released by the synovium. (c) Constructed synergistic cytokine network that regulates ECM remodeling in chondrocytes (left panel). Numbers in the network represent the number of studies supporting the synergistic effect (*p*‐value <0.05). Results of clinical trial (NCT02477059) targeting IL6 is embedded in the network for illustration purpose. Ligand‐receptor complex of IL6 as well as OSMR are also provided for illustration purpose. (d) Predicted OSM level was significantly associated with injury‐induced transcriptomic response of ECM remodeling genes. (e) Trajectory of *Osmr* gene expression after ACL rupture. (f) *Tabula Muris Senis* database revealed that while *Osm* expression was predominantly driven by neutrophils, *Osmr* expression was predominantly driven by chondrocytes. (g) Schematic illustrating possible neutrophil–chondrocyte interaction that activates the OSM‐OSMR axis in aged murine knee joints after mechanical overload. Statistical analyses were performed using linear regression (d). Portions of the figures were created with Biorender.com. ACL, anterior cruciate ligament; ECM, extracellular matrix; PC, principal component.

We next tested for candidate drivers of the observed aberrant ECM remodeling with aging. Accumulating evidence has shown that ECM remodeling is induced by inflammatory cytokines (Wynn & Ramalingam, 2012). Traumatic knee injuries trigger an immediate increase of inflammatory cytokines in the synovial fluid (Lieberthal et al., 2015). As inflammatory synovial cytokines disturb joint homeostasis and are implicated in the pathophysiology of KOA (Kapoor et al., 2011), we explored cytokines released exclusively by the synovium as potential upstream regulators for the age‐related aberrant ECM remodeling in the aged knee after injury (Figure 4a). Using the 58 genes related to ECM remodeling identified above as inputs (Figure 2d,e), CytoSig analysis (Jiang et al., 2021) identified a total of 43 cytokines, in which 12 cytokines were predicted to be elevated in the aged knee joint after ACL rupture relative to young counterparts (Figure 4a). Of these 12 cytokines, OSM and IL6 were predominantly expressed in synovial cells but not in chondrocytes (<1%), according to single‐cell RNA‐Seq data of articular cartilage and synovium isolated from elderly individuals with KOA (GSE152805) (Chou et al., 2020) (Figure 4b).

IL6 and OSM are members of the IL6 family of cytokines and are produced by immune cells in response to tissue injury (Rose‐John, 2018). IL6 signaling is actively involved in regulating cartilage integrity and pain, implicating IL6 as a potential therapeutic target for murine post‐traumatic knees (Liao et al., 2022). However, a recently conducted clinical trial of IL6 receptor antagonist, tocilizumab, showed no significant pain relief in older subjects with hand OA when compared to placebo (Richette et al., 2021). OSM is a promising alternative to IL6 agonists because OSM contributes to the network of cytokines, mediators, enzymes, and structural proteins that control ECM homeostasis, which are unique to OSM among the IL6 family (Richards, 2013). OSM primes and amplifies inflammatory responses to IL1, TNF‐α, or IL17A in the joint (Richards, 2013), all of which lead to compromised cartilage integrity. Indeed, when we originally defined the synergistic network of cytokines surrounding to OSM and IL6 in regulating cartilage ECM remodeling (e.g., TIMPs and MMPs) through systematic literature search, we found that OSM produced synergistic effects with other cytokines in regulating the cartilage ECM remodeling process (Figure 4c). Full information for the synergistic effects of cytokines is available in Table S5. Further supporting the regulatory role of OSM, the predicted OSM level was positively associated with age‐related remodeling genes (Figure 4d).

While OSM and IL6 have redundant biological functions due to the common signal transducing receptor, gp130, OSM also produces nonredundant signals through its unique receptor, OSMR (Rose‐John, 2018). As signals downstream of OSMR mediate OSM‐induced ECM remodeling (Stawski & Trojanowska, 2019), we revisited the individual transcriptomic responses of the archived RNA‐Seq data (Sebastian et al., 2020). We found that OSMR was significantly increased in aged mice up to 2 weeks after ACL rupture (false discovery rate <0.001; Figure 4e), displaying a trajectory similar to the predicted OSM level. These findings are in line with previous studies demonstrating that patients with KOA displayed increased OSM concentration in synovial fluid and that osteoarthritic cartilage displayed increased OSMR levels (Beekhuizen et al., 2013; Tsuchida et al., 2014). Notably, a KOA‐related increase in OSM within the synovial fluid inhibits ECM synthesis by chondrocytes, an effect that was counteracted by inhibiting OSM (Beekhuizen et al., 2013). Although OSM also binds to leukemia inhibitory factor receptor (LIFR) according to the comprehensive ligand‐receptor database, CellChat (Jin et al., 2021), LIFR was not detected in the RNA‐Seq data we analyzed (Sebastian et al., 2020). Together, these findings indicate that OSM may be a driver of pathogenic ECM remodeling in aged cartilage via the OSM–OSMR axis.

In light of the catabolic effect of OSM–OSMR shown in previous studies, we next sought to determine the cellular origin of these age‐dependent responses to mechanical overloading. For this purpose, we accessed the *Tabula Muris Senis* database (Tabula Muris Consortium; Overall Coordination; Logistical Coordination; Organ Collection and Processing; Library Preparation and Sequencing; Computational Data Analysis; Cell Type Annotation; Writing Group; Supplemental Text Writing Group; Principal Investigators, 2018) to assess which cells predominantly express OSM and OSMR in murine tissue. Results revealed that, whereas OSM is predominantly expressed in immune cells, and especially neutrophils, OSMR is predominantly expressed in chondrocytes (Figure 4f). These findings from murine studies are in line with the human study which demonstrated that, while OSM is highly expressed in HLA‐DRA^+^ cells (including immune regulatory cells, inflammatory macrophages, dendritic cells, activated proinflammatory fibroblasts, and B cells), OSMR is highly expressed in chondrocytes in the synovium and/or cartilage samples in people with KOA (Chou et al., 2020). Neutrophils are the first immune cells to be recruited in the inflamed joint, where they secrete proinflammatory mediators (Li et al., 2022). As articular cartilage is comprised exclusively of chondrocytes, these data indicate neutrophil‐to‐chondrocyte interactions emanating from the synovium and cartilage, respectively. This potential neutrophil‐to‐chondrocyte interaction is in line with a previous study demonstrating that patients with KOA displayed increased neutrophil‐related inflammation in the synovial fluid (Haraden et al., 2019; Manukyan et al., 2023). Exaggerated neutrophil recruitment contributes to the development of tissue damage and fibrosis (Wynn & Ramalingam, 2012), which may be partly triggered by IL‐17 (Laan et al., 1999), one of the cytokines significantly predicted by our cytokine inference analysis (Figure 4a). Indeed, a recent study showed that IL‐17 levels in inguinal lymph nodes of aged mice after traumatic knee injury were significantly higher than those in young mice, while IL‐17 neutralization reduced traumatic injury‐induced cartilage degeneration in aged mice (Faust et al., 2020). Taken together, these data led us to the novel hypothesis that a trauma‐induced increase in OSM from the synovium binds to OSMR in chondrocytes, initiating downstream signal activation, age‐related aberrant ECM remodeling, and ultimately, chondrocyte dysfunction (Figure 4g).

### 2.5 In silico and in situ activation of the OSM–OSMR axis induced aberrant ECM remodeling in aged chondrocytes

**FIGURE 5:**
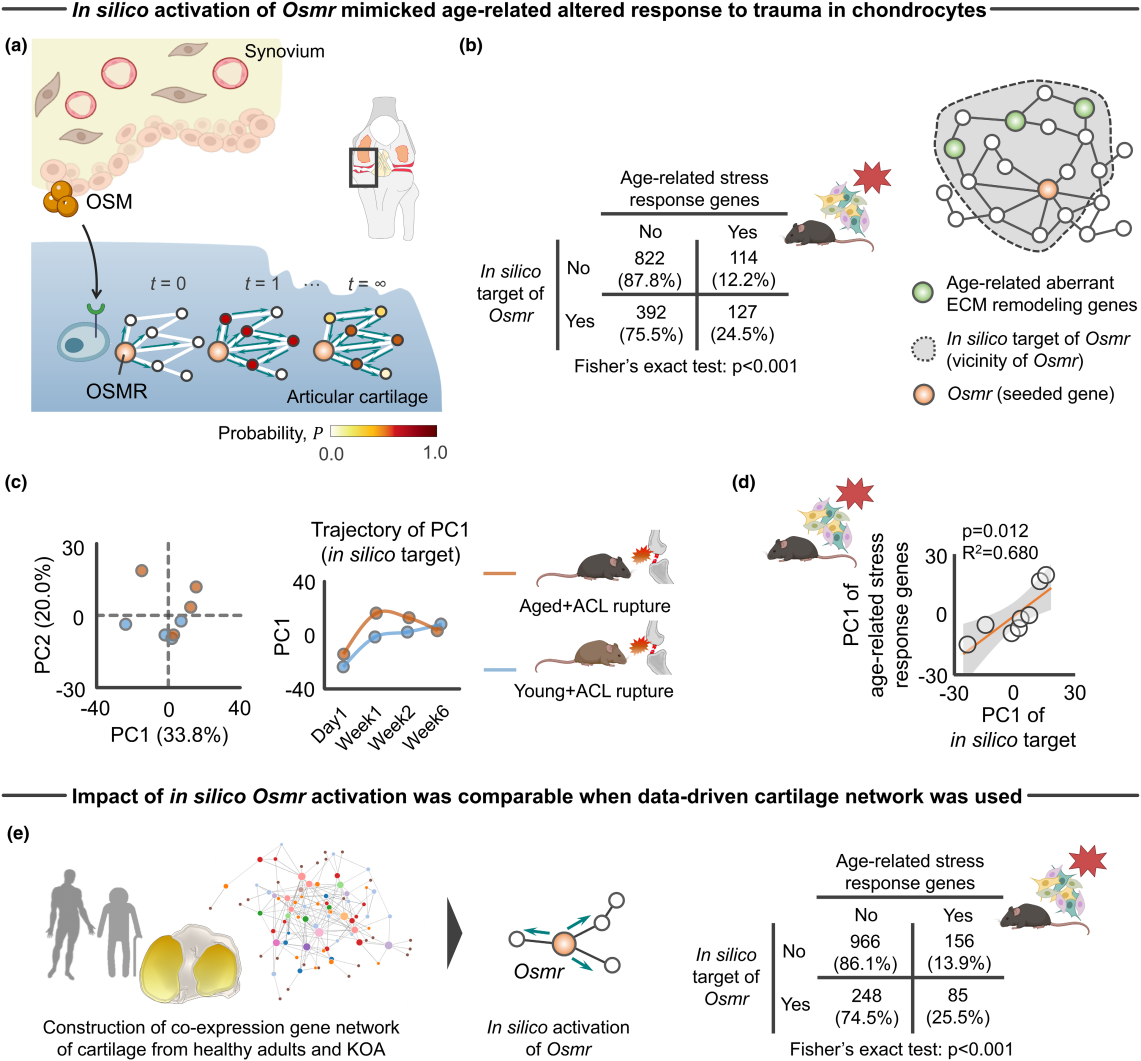
In silico network approach predicting the impact of pseudo activated OSM–OSMR axis for cartilage‐specific network. (a) Schematic showing tissue‐specific network propagation that simulates tissue‐tissue interaction of synovium (OSM) and cartilage (OSMR) in knee joints. (b) In silico target genes of *Osmr* displayed significant enrichment to age‐related stress response genes. Values in the 2 × 2 contingency table show number of genes (percentage). (c) PCA showing the separate trajectory of time course changes in transcripts response of in silico target genes of *Osmr* to ACL rupture between young and aged mice. (d) Transcriptomic response of in silico target genes of *Osmr* was associated with age‐related stress response genes. (e) Sensitivity analysis of in silico activation of *Osmr* on the different co‐expression networks constructed from cartilage samples of people with healthy adults and KOA. WGCNA (Langfelder & Horvath, 2008) was used to construct the network according to bulk RNA‐Seq data (GSE114007) (Fisch et al., 2018).Values in the 2 × 2 contingency table show number of genes (percentage). Statistical analyses were performed using Fisher's exact test (b and e) or linear regression (d). Portions of the figures were created with Biorender.com. ACL, anterior cruciate ligament; ECM, extracellular matrix; KOA, knee osteoarthritis; PC, principal component; PCA, principal component analysis; WGCNA, weighted gene correlation network analysis.

To test the above hypothesis suggested, we implemented network propagation applied to the cartilage‐specific gene network. This approach is based on the premise that network propagation represents tissue interaction of OSM (released by synovium) and OSMR (chondrocyte in articular cartilage) (Figure 5a). Network propagation explores the network vicinity of seeded genes to study their functions based on the premise that nodes with similar functions tend to lie close to each other in the networks (Cowen et al., 2017). To simulate, we first constructed a knowledge‐driven global cartilage‐specific network using HumanBases software (Greene et al., 2015). With this global network in hand, we posited that in silico activation of *Osmr* in chondrocytes would activate the age‐related stress response genes we found above to be overexpressed with trauma in aged joints. For this purpose, a random‐walk‐restart (RWR) algorithm (Valdeolivas et al., 2019) was used to verify which nodes are most frequently visited on a random path in a given network. As expected, RWR applied to cartilage‐specific network revealed that genes pseudo‐activated (i.e., an affinity score > 0) by *Osmr* were significantly associated with age‐related stress response genes (Figure 5b). This finding suggests that age‐related stress response genes are in the vicinity of *Osmr* and, therefore, age‐related aberrant ECM remodeling, a primary functional module of age‐related stress response genes, are functionally connected with *Osmr* in regulating cartilage homeostasis.

To compare the transcriptomic response of in silico target genes of *Osmr* with age‐related stress response genes against traumatic injury, we revisited archived RNA‐Seq data (Sebastian et al., 2020) and performed PCA with the transcriptomic response of in silico target genes included as input. PCA revealed that the injured aged knee joint displayed a distinct transcriptomic response to in silico perturbation of *Osmr* target genes when compared to young counterparts (Figure 5c). Intriguingly, the transcriptomic response of in silico target genes of *Osmr* was significantly associated with age‐related stress response genes (Figure 5d), supporting the hypothesis that activated OSM–OSMR axis recapitulates an age‐related transcriptomic response to injury and induces aberrant ECM remodeling in aged cartilage.

To address the possibility that the effects of in silico activation on age‐related stress response genes is a function of type of network constructed (i.e., knowledge‐driven vs. data‐driven), we also built the data‐driven gene co‐expression network based on the transcriptomic response of human cartilage (GSE114007) (Fisch et al., 2018). The data‐driven de novo networks indicate putative biomolecular interactions within a specific biological condition and help to elucidate disease networks and predict therapeutics in a more holistic way (Rintala et al., 2022). Weighted gene correlation network analysis (WGCNA) is a data‐driven approach to generate gene–gene co‐expression networks from all pairwise correlations (Langfelder & Horvath, 2008). Consistent with findings from the original network constructed by HumanBases (Figure 5a,b), pseudo‐activated genes by in silico *Osmr* activation were significantly associated with age‐related stress response genes (Figure 5e).

**FIGURE 6:**
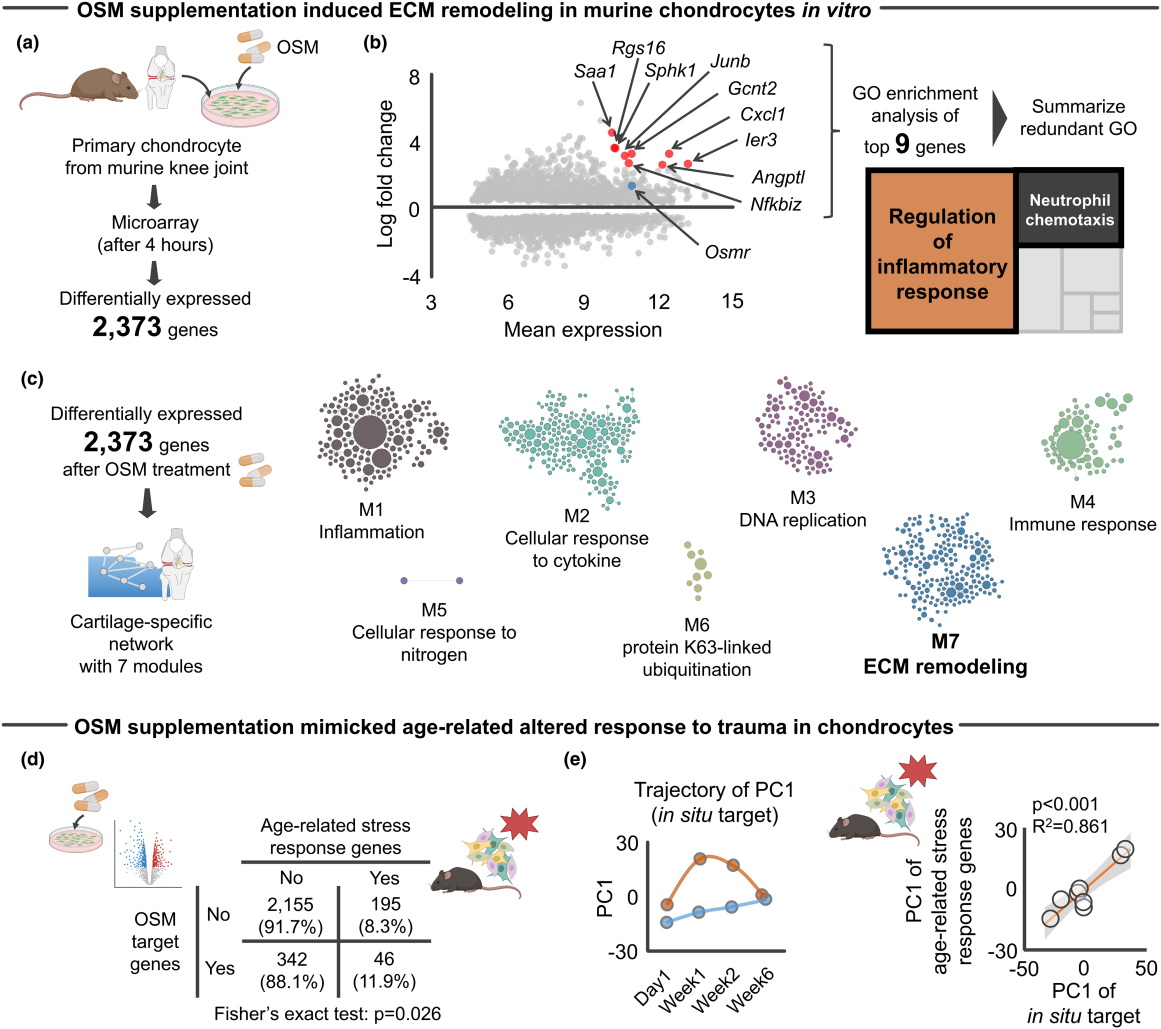
OSM influenced age‐related aberrant ECM remodeling genes in murine chondrocytes. (a) Schematic showing the experimental flow of microarray data ± OSM supplementation to primary murine chondrocytes (Liu et al., 2015). The microarray revealed 2373 differentially expressed genes at 4 h after OSM supplementation to chondrocytes. (b) GO enrichment analysis with subsequent summarizing redundant GO of top nine genes significantly upregulated (mean expression >10, log fold change >2.5) identified inflammation‐related GO biological functions, such as “Regulation of inflammatory response” and “Neutrophil chemotaxis.” In the MA plot, *Osmr* is highlighted with blue color for a descriptive purpose. (c) Cartilage‐specific network constructed based on 2373 differentially expressed genes. The second largest module (module7: M7) was annotated as “ECM remodeling.” (d) Association between differentially expressed 2373 genes after OSM supplementation and age‐related stress response genes. Values in the 2 × 2 contingency table indicate the number of genes (percentage). (e) PCA showing the separate trajectory of time course changes in transcripts response of in situ target genes of OSM to ACL rupture between young and aged mice. Transcriptomic responses of in situ target genes of OSM were associated with those age‐related stress response genes. Statistical analyses were performed using Fisher's exact test (d) or linear regression (e). Portions of the figures were created with Biorender.com. ACL, anterior cruciate ligament; ECM, extracellular matrix; GO, gene ontology; PC, principal component; PCA, principal component analysis.

In addition to the in silico analysis, we also sought to show direct evidence of OSM on age‐related aberrant ECM remodeling in situ. We accessed archived microarray data of primary chondrocytes that were isolated from murine knee joints with or without OSM supplementation (Liu et al., 2015) (Figure 6a). The original study by Liu et al was designed to compare transcriptomic response of articular chondrocytes to treatments by IL6 family cytokines (OSM, IL6, IL11, or leukemia inhibitory factor; 100 ng/mL in each cytokine) (Liu et al., 2015). The results showed that OSM supplementation significantly changed the expression level of 2373 genes, among which OSMR was significantly upregulated after treatment (Figure 6b). Of note, among the 2373 genes identified, nine genes were highly upregulated (mean expression >10, log fold change >2.5). The nine upregulated genes were significantly associated with inflammation‐related biological function such as “Regulation of inflammatory response” and “Neutrophil chemotaxis” (Figure 6b), which is in line with our findings that OSM was predominantly released by neutrophils (Figure 4f).

We then determined the biological function of 2373 differentially expressed genes using a cartilage‐specific network and identified an ECM remodeling module (Figure 6c). Most notably, the differentially expressed genes after OSM supplementation were significantly associated with age‐related stress response genes (Figure 6d). Further, similar to our in silico prediction (Figure 5c,d), the aged knee joint displayed different transcriptomic responses of *Osmr* target genes in situ (Figure 6e), which were also significantly associated with age‐related stress response genes (Figure 6e). As the original study by Liu et al demonstrated the divergent transcriptomic response among the IL6 family cytokines (Liu et al., 2015), we additionally accessed archived microarray data of primary chondrocytes that were isolated from murine knee joints with or without IL6 supplementation (Liu et al., 2015) and asked whether the observed age‐related stress response after OSM supplementation is driven by a unique biological function of OSM that is independent from IL6, another cytokine predicted to be a possible driver of the aberrant ECM remodeling (Figure 4b). We found that the genes significantly regulated by OSM, but not IL6, were significantly associated with age‐related stress response genes (Figure S9). Interestingly, this significant relationship was not confirmed in genes that were significantly regulated by IL6 (Figure S9), indicating that OSM‐induced age‐related stress response after trauma in chondrocytes is predominantly driven by genes uniquely responsive to OSM.

### 2.6 In silico and in situ activation of the OSM–OSMR axis recapitulated an inflammatory phenotype of people with KOA

**FIGURE 7:**
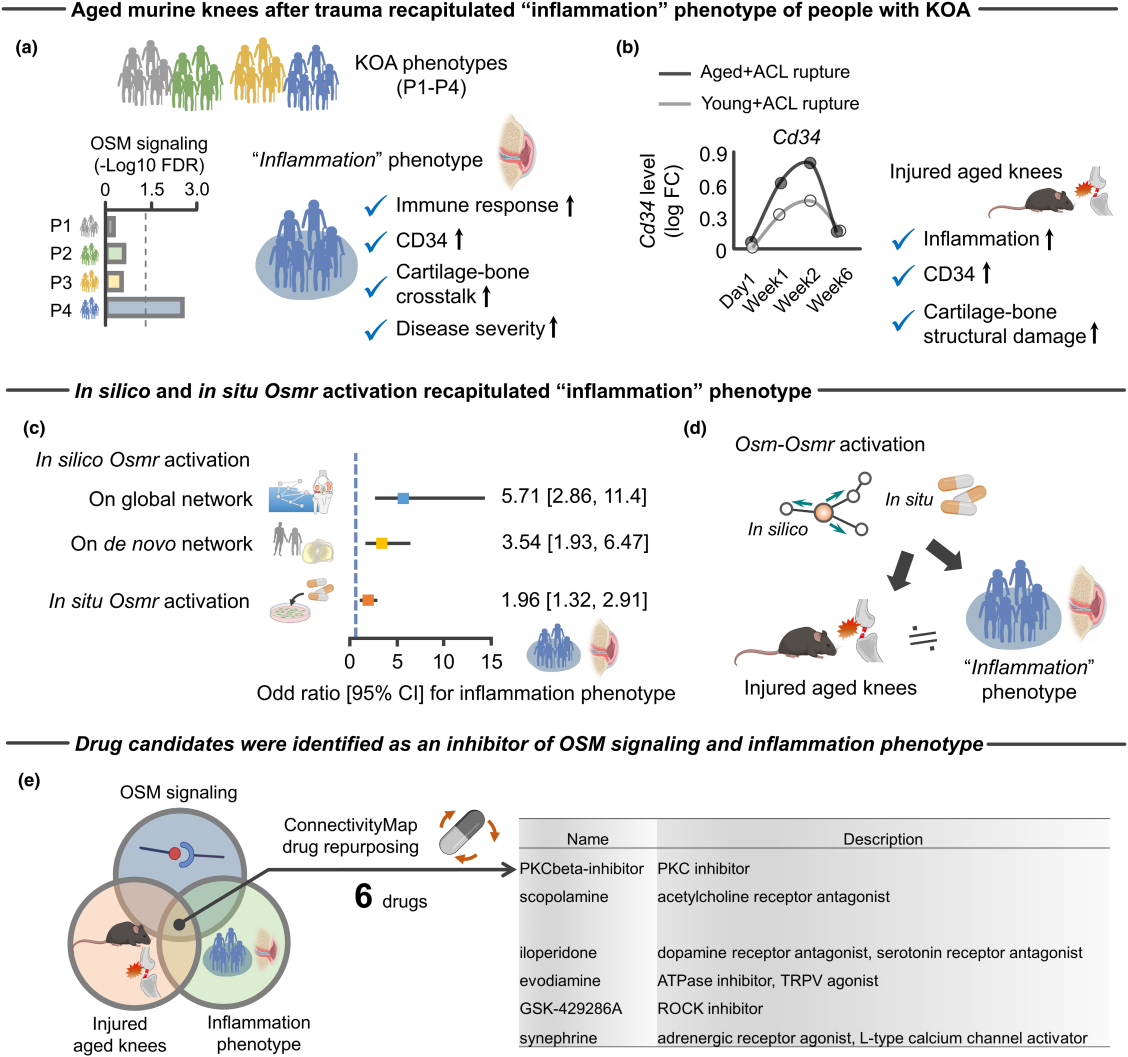
Injured aged murine knees and activation of *Osmr* recapitulated “inflammation” KOA phenotype. (a) Schematic showing the molecular and clinical features of an “inflammation” phenotype, a one of the four phenotypes (P1–P4) of people with KOA that are defined by previous study (Yuan et al., 2020). Only the inflammation phenotype displayed significant enrichment to OSM signaling pathway. P1, GAG metabolic disorder; P2, collagen metabolic disorder; P3, activated sensory neurons; P4, inflammation. (b) Aged mice after ACL rupture displayed increased *Cd34* gene expression compared to young counterparts. Injured aged murine knees have a similar molecular (inflammation and CD34) and structural (cartilage‐subchondral bone damage) profiles seen in the inflammation phenotype of people with KOA. (c) In silico and in situ target genes of *Osmr* displayed significant enrichment to genes associated with the inflammation phenotype (i.e., odd ratio in each model is over 1). Results were similar across the two different in silico networks model (cartilage‐specific network constructed by Humanbases (Greene et al., 2015) and de novo co‐expression network constructed by WGCNA according to archived RNA‐Seq data (GSE114007) (Fisch et al., 2018)). The blue dotted line represents an odd ratio with 1. (d) Schematic summarizing the findings that the injured aged murine knees and activation of *Osmr* recapitulated “inflammation” KOA phenotype. (e) Six compounds with the strongest evidence for inducing gene expression signatures that counter elevated Oncostatin M signaling, upregulated age‐related stress response genes, and inflammation phenotype. All of these compounds were provided by ConnectivityMap (Subramanian et al., 2017). Statistical analyses were performed using binary logistic regression analysis (c). 95% CI, 95% confidence interval; ACL, anterior cruciate ligament; FC, fold change; FDR, false discovery rate; GAG, glycosaminoglycan; KOA, knee osteoarthritis; OSM, Oncostatin M; OSMR, Oncostatin M receptor; WGCNA, weighted gene correlation network analysis.

Finally, we sought to correlate the findings from the murine studies with pathophysiology of KOA. Since KOA is a complex heterogeneous disease with multiple etiologies, recent studies have been dividing KOA into different phenotypes (Mobasheri et al., 2019). Among the suggested phenotypes (“GAG metabolic disorder,” “collagen metabolic disorder,” “activated sensory neurons,” and “inflammation”), much interest has been directed to “inflammation” phenotype because of therapeutic potential by anti‐inflammatory drugs (Cao et al., 2021). Using single‐cell RNA‐Seq approach of human osteoarthritic cartilage, a recent study characterized the inflammation phenotype as an elevated immune response, upregulated CD34, increased cross talk of synovium‐cartilage‐subchondral bone, and severe disease severity (Yuan et al., 2020) (Figure 7a). In addition to these findings from the original study, we found that the inflammation phenotype displays significantly higher enrichment to OSM signaling pathway (Figure 7a). These clinical and biological features are shared in injured age murine knees, as evidenced by elevated inflammation, structural damage of cartilage‐subchondral bone, and increased *Cd34* expression compared to young counterparts (Figure 7b). The similarities suggest that aged murine knees after traumatic injury recapitulate the inflammatory phenotypes of people with KOA.

We further simulated whether *Osmr* is a possible driver of pathophysiology of the inflammation phenotype. Using the identified set of genes (1721 genes) that characterized the inflammation phenotype as a reference (Yuan et al., 2020), we again repeated in silico and in situ activation of *Osmr*. Consistent with the close relationship between injured aged murine knees and inflammation phenotype, *Osmr* activation significantly changed expression levels of genes associated with inflammation phenotype (Figure 7c). Taken together, these findings suggest that injured aged murine knees recapitulate pathophysiology of an inflammatory phenotype of human KOA, in which elevated OSM‐OSMR axis may be a possible cytokine driver (Figure 7d).

#### 2.6.1 Identification of potential drugs to attenuate OSM signaling and inflammation phenotype

Having molecular similarities across OSM signaling, injured aged knees, and an inflammatory phenotype in KOA, we sought to identify compounds capable of reversing these changes. Using in vitro drug screen data from ConnectivityMap (Subramanian et al., 2017), we identified six compounds that induce strong opposing gene expression signature across the targets (i.e., OSM signaling, injured aged knees, and inflammation phenotype), attenuating the disease burden (Figure 7e). Some of these compounds have known biological relevance to pathophysiology of KOA, including TRPV agonist and ROCK inhibitor (Kim et al., 2015; Lv et al., 2021) (Figure 7e). Intriguingly, ROCK is a known regulator of ECM remodeling (Kim et al., 2015), consistent with our findings of age‐related aberrant ECM remodeling and stiffening as a downstream of OSM signaling (Figures 4b and 5e).

Further notable examples of compounds with the potential to reverse pathological molecular changes included dopamine receptor antagonist that has therapeutic potential and attenuate IL‐17‐mediated neutrophilic airway inflammation (Nakagome et al., 2011). Other compounds, including PKCβ inhibitor and adrenergic receptor agonist, also have anti‐inflammatory effects (Ağaç et al., 2018; Alleboina et al., 2020), serving as a possible new strategy for treating inflammation phenotype of KOA. These compounds may have a candidate role in preventing the onset and/or progression of inflammation phenotype of KOA.

## 3 DISCUSSION

An age‐related decline in intrinsic chondrocyte repair responses to traumatic injury has been suggested to accelerate the progression of KOA (Houtman et al., 2021; Loeser, Olex, et al., 2012; Madej et al., 2016). With aging of the global population and the expanding prevalence of KOA, there is a growing need to understand how aging and trauma‐induced stress interact to accelerate KOA. To address this critical issue, this study introduced a network paradigm of cytokine inference integrated with network propagation in a cartilage‐specific network (see Graphical Abstract). Using this approach, we identified the OSM‐OSMR axis as a previously unappreciated cytokine driver of age‐related aberrant ECM remodeling, possibly via intercellular communication of synovial neutrophils and articular chondrocytes. The aberrant ECM remodeling was accompanied by transcriptomic responses that induce ECM stiffening. These age‐related altered transcriptomic responses are in line with tissue level changes, as evidenced by our meta‐analysis demonstrating that aged knee joints display accelerated compromised cartilage integrity following injury when compared to young counterparts. Intriguingly, while OSM is an IL6 family cytokine, pharmacological manipulations suggested that the age‐related aberrant ECM remodeling is attributed, at least partly, to downstream signals of OSM, but not IL6. The molecular and tissue level changes in injured aged knees recapitulate the pathophysiology of inflammation phenotype in people with KOA, in which the OSM‐OSMR axis is a possible cytokine driver and potential novel target for the development of new therapies. The subsequent drug repurpose was built upon this theme and identified six molecules with potential clinical application to attenuate the OSM–OSMR axis as well as inflammation phenotype in people with KOA.

A critical knowledge gap in the investigation of age‐related accelerated KOA has been the lack of characterization of an age‐specific signature of the transcriptomic response to a traumatic injury. With the identified stress response genes uniquely upregulated in aged knee joints, this study implemented cartilage‐specific network analysis. Results revealed that aberrant remodeling and stiffening of the ECM after trauma is an age‐specific feature. ECM remodeling is a widely recognized cause of ECM stiffening (Bonnans et al., 2014). In turn, ECM stiffening is a known driver of chondrocyte dysfunction and disrupted cartilage integrity via mechanotransductive pathways, both in the post‐traumatic KOA in young mice (Kim et al., 2015) as well as naturally occurring KOA in aged mice (Iijima et al., 2023). Studies have shown that traumatic injury in young murine knee joints display altered transcripts associated with ECM remodeling and induced ECM stiffening via LOX‐mediated increased collagen cross‐linking (Kim et al., 2015; Sebastian et al., 2020). Findings of the current study suggest that aging amplifies the trauma‐induced ECM remodeling and stiffening, driving an accelerated disease process.

A separate cartilage‐specific network analysis for stress response genes uniquely downregulated in aged knee joints revealed that an altered oxidative stress response is also an age‐specific feature. Oxidative stress, which refers to an imbalance between the production of reactive oxygen species and the ability to repair the resulting damage, has been implicated in aberrant ECM remodeling and in the pathogenesis of KOA (Loeser et al., 2016). For example, oxidative stress impacts cartilage ECM integrity via alterations in the function of key molecules such as MMPs (Loeser et al., 2016). Integrating these previous reports with our current findings from the cartilage‐specific network analysis suggests that aberrant ECM remodeling in aged knees after traumatic injury may be attributed, at least in part, to reduced resistance to oxidative stress. Additional efforts to clarify the intricate interplay between oxidative stress and ECM remodeling in aged cartilage after traumatic injury is crucial for developing targeted therapeutic strategies that will support the preservation of cartilage health over time.

In these analyses, the utility of implementing the cartilage‐specific network analysis is supported by our finding that neither traditional GO enrichment analysis nor non‐cartilage‐specific network identified ECM remodeling as a primary biological function altered in aged knee joints. The tissue‐specific networks used in the current study were developed as genome‐scale functional maps of human tissues by integrating a collection of data sets integrating thousands of experiments contained across more than 14,000 distinct publications (Greene et al., 2015). As such, implementing the cartilage‐specific network allowed us to profile the specialized function of genes in a high‐throughput manner in the context of cartilage biology. It should be emphasized that, owing to the low chondrocyte density (~2% of the total volume of cartilage (Alford & Cole, 2005)) and the lack of blood supply in articular cartilage, chondrocyte behavior largely depends on input signals from surrounding ECM (van der Kraan et al., 2002). This tissue feature is unique to articular cartilage and emphasizes the need for implementation of tissue‐specific network in inferring the cellular response to external environmental perturbations. The consideration of tissue specificity is much needed in the context of aging research, particularly given accumulating evidence showing that aging induces transcriptomic changes in a tissue‐specific manner (Wang et al., 2022; Yamamoto et al., 2022).

The development and progression of KOA are now believed to involve inflammation even in the early stage of the disease (Mahmoudian et al., 2021). Synovial cells coordinate the production of molecules that initiate and maintain synovial inflammation and contribute to cartilage damage in the setting of OA (Sanchez‐Lopez et al., 2022). Among the proinflammatory cytokines involved in KOA, IL‐1β, and TNFα have been considered to be major regulators of cartilage degeneration (Wojdasiewicz et al., 2014). Indeed, aged mouse knee joints displayed prolonged suppression of proteoglycan synthesis after IL‐1β exposure when compared to young mice (van Beuningen et al., 1991). However, the results of KOA clinical trials conducted to date, which have mostly studied the effect of anti‐IL1β and anti‐TNF therapies, have yielded disappointing results (Latourte et al., 2020). Beyond IL‐1β and TNFα, other cytokines are being investigated as targets of treatment for KOA, including IL6. IL6 targets JAK/STAT signaling, which has been implicated in the pathogenesis of inflammatory and autoimmune diseases including rheumatoid arthritis (Banerjee et al., 2017). However, as was observed for IL‐1β and TNFα, a recent clinical trial (NCT02477059) targeting anti‐IL6 also showed no significant pain relief effect in patients with hand OA (Richette et al., 2021). Using network‐based cytokine inference integrated with network propagation that simulates synovium‐cartilage cross talk, the current study identified the OSM–OSMR axis as a predicted driver of the aberrant ECM remodeling that we found to be unique to the aged knee joint after injury. Intriguingly, pharmacological manipulation revealed that OSM–OSMR axis induces differential transcriptomic responses in chondrocytes as compared to IL6. Moreover, the genes uniquely regulated by OSM–OSMR were significantly associated with age‐related aberrant ECM remodeling after traumatic injury. Secreted OSM binds its unique receptor, OSMR, which, in turn, activates downstream JAK/STAT signaling pathway to promote ECM remodeling and senescence of cartilage‐derived stem/progenitor cells (Ji et al., 2022; Li et al., 2001; Stawski & Trojanowska, 2019). While, to our knowledge, no clinical study in KOA has been designed to target OSM, a new generation of anti‐OSM antibody, GSK2330811, was recently developed and tested in a clinical trial for the treatment of systemic sclerosis (Denton et al., 2022).

Our network‐based cytokine inference also identified IL17A as a possible driver cytokine for age‐related aberrant ECM remodeling and stiffening. As indicated by the synergistic cytokine network we defined, IL17 acts an amplification factor together with OSM for other inflammatory cytokines (Richards, 2013; Wang et al., 2022). IL17A activates neutrophils through production of IL8, a key chemokine for neutrophils (Wang et al., 2022). Given that OSM originates from neutrophils, as indicated by *Tabula Muris Senis*, IL17A may be upstream of OSM release by neutrophils. While this IL17A‐OSM (neutrophil) axis has been traditionally implicated in the pathogenesis of rheumatoid arthritis (Wang et al., 2022; Wright et al., 2014), evidence has suggested it may also play a role in the onset of KOA (Haraden et al., 2019; Manukyan et al., 2023; Mimpen et al., 2021). For example, IL17A induces chondrocyte senescence and trauma‐induced KOA in aged murine knees (Faust et al., 2020). Another study showed that neutrophils may constitute a population of as much as 25% of the cells in the synovial fluid in patients with KOA (Hsueh et al., 2021; Kriegová et al., 2018). Neutrophil elastase in synovial fluid induces chondrocyte apoptosis and activates the caspase signaling in OA (Wang et al., 2021). Further, IL17A significantly amplifies OSM‐induced production of ECM degradation enzymes in human cartilage explant and chondrocyte (Moran et al., 2009), suggesting that therapeutics targeting IL17A together with OSM may be a promising approach to prevent or delay trauma‐induced KOA in aged knees. Preclinical and subsequent clinical studies targeting the synergistic effects of these cytokines represent an interesting future direction.

The findings of the current study have important clinical implications for the development of novel therapeutics. Correlating the downstream effects of OSM–OSMR axis with transcriptomic signature of phenotypes in people with KOA, we discovered that activation of the OSM–OSMR axis recapitulated an inflammatory phenotype of KOA and identified high‐value targets for drug development and repurposing. These findings offer translational opportunities targeting the inflammation‐driven KOA phenotype and bring us one step closer toward establishing phenotypic drug discoveries for people with KOA.

Although this study provides a new perspective to the pathogenesis of age‐related KOA, it has limitations. First, our meta‐analysis for age‐related accelerated KOA was based on a small number of studies, which may contribute to bias depending on the methodology used in the original studies. For example, we cannot discount the possibility that the different experimental models of trauma included (i.e., DMM, ACL rupture, and compressive loading) may trigger distinct molecular and cellular responses, even if disease severity appears similar across the groups. It should be noted that the OSM–OSMR axis identified was driven by RNA‐Seq data from cartilage following an ACL rupture. As such, future studies should seek to evaluate the robustness of the findings while considering other experimental models (e.g., DMM and excessive compressive loading) and subsequent validation with clinical samples. Additionally, given the lack of female mice in current studies, we were not able to clarify mechanisms underlying age‐related KOA in female mice, and this remains an important area for future work. Second, findings of this study were predominantly obtained from the C57BL6 mice strain. Therefore, we cannot exclude the possibility that OSM–OSMR axis as a driver of ECM remodeling may be strain specific. Finally, we constructed a protein–protein interactive network based on information obtained from gene knockout experiments to visualize functional relationship of genes contributing to cartilage ECM stiffening in murine knee joint. However, these results should be interpreted with a caution given that information from gene knockout experiments may have discordance with protein function owing to the lack of consideration for posttranslational modification, alternative splicing, and protein complex formations (Baralle & Giudice, 2017; Duan & Walther, 2015).

While this study focused on KOA as a model, a major conceptional innovation of our work is the network‐based cytokine inference integrated with network propagation on cartilage‐specific network to simulate signal transduction in cartilage as a downstream of synovium‐derived cytokines. It is widely recognized that age‐related, low‐grade inflammation—“inflammaging”—contributes to the pathogenesis of age‐related diseases via chronic activation of the innate immune system involving inflammatory cytokines (Franceschi et al., 2018). As such, we anticipate that the introduced network approaches use here may have broader applications in the field of aging research, even beyond KOA.

## 4 MATERIALS AND METHODS

### 4.1 Systematic literature search for age‐related alterations to mechanical overloading

The systematic literature search was conducted according to the Preferred Reporting Items for Systematic reviews and Meta‐Analyses (PRISMA) statement (Moher et al., 2009), PRISMA protocols (PRISMA‐P) (Shamseer et al., 2015), Meta‐analysis of Observational Studies in Epidemiology (MOOSE) checklist (Stroup et al., 2000), Cochrane Handbook for Systematic Reviews of Interventions (Higgins & Green, 2011), and the practical guide for meta‐analysis from animal studies (Vesterinen et al., 2014).

Manuscript eligibility criteria were defined according to the PICO (P, patient; I, intervention; C, comparison; O, outcome) (Methley et al., 2014). In brief, we included articles characterizing age‐related alterations in articular cartilage and/or subchondral bone of murine knee joints to traumatic injury (i.e., each study had to include both aged and young mice or middle‐aged and young mice). Young, middle‐aged, and aged mice were defined as 2–7, 9–15, and 18–24 months old, respectively, and correspond to ages 20–30, 38–47, and 56–69 years in humans, respectively (Jackson et al., 2017). As middle‐aged and aged mice display similar proteomic signatures (Iijima et al., 2023), this study combined middle‐aged and aged mice and defined them as the “aged” group. No restrictions were set according to mouse strain or article publication year. The following articles were excluded: (1) studies that included genetically modified animals, as such models likely oversimplify the disease process, whereas naturally occurring OA is almost certainly polygenic in nature (Little & Hunter, 2013); (2) studies that did not explicitly compare articular cartilage or subchondral bone in the knee joint between young and aged mice; and (3) treatment arm includes another intervention (e.g., saline intra‐articular injection), which may interact with aging effects on the disease progression.

Outcome measurements related to assessment of articular cartilage damage or subchondral bone alterations were divided into six categories based on a slightly modified version of previous meta‐analyses (Bricca et al., 2019; Rongen et al., 2015). We used the following hierarchy of outcomes:

1. morphology (e.g., Osteoarthritis Research Society International [OARSI] score (Pritzker et al., 2006), Mankin's score (Mankin et al., 1971), MRI‐based classifications of morphological changes);
2. morphometry (any kind of quantitative methods on microscopic images of articular cartilage and subchondral bone including computational image analysis techniques (Moussavi‐Harami et al., 2009));
3. composition (any kind of quantitative methods for quantification of proteoglycan or collagen);
4. biomechanical characterization (e.g., tensile and compressive measures of stiffness);
5. biomarker (e.g., mirco RNA in synovial fluid);
6. molecular biology (e.g., ECM‐related gene expression and protein synthesis)

PubMed was used for the electronic database search. Google Scholar was also used as a complementary search engine. A manual search of the reference lists of past reviews was performed (Jørgensen et al., 2017). Furthermore, citation searching was performed on the original records using Web of Science. These citation indexes are recommended by the Cochrane Handbook (Higgins & Green, 2011). For the database search was performed on 14 November 2022. Electronic searches for PubMed used combined key terms, including “Animal, Laboratory” “Aging,” “Age factors,” “Cartilage, Articular,” and “Osteoarthritis” using Medical Subject Headings terms.

A single reviewer (HI), as a content and statistical expert, assessed eligibility in accordance with the Cochran Handbook recommendations (Higgins & Green, 2011). The reviewer screened titles and abstracts yielded by the search. Full manuscripts of the articles that met the eligibility criteria were then obtained and reviewed. During these processes, the reviewer prepared and used simple, predesigned Google spreadsheets to assess eligibility by extracting study features.

The same reviewer extracted data regarding basic study information (authors, publication year, and country of corresponding author), experimental condition (i.e., mice strain, age, sample size, and sex), target joint (tibiofemoral or patellofemoral joints), and outcome measures. If outcome measures from multiple time points were reported within the same age category (e.g., 3 and 6 months old from the young group), we averaged the effect size (López‐López et al., 2018). If outcome measures from multiple compartments (e.g., medial and lateral compartments in tibiofemoral joint and patellofemoral joint) were presented, data from the most severe region were extracted. When data were not reported or were unclear, we contacted the authors directly. A reminder was sent to those who had not replied. If data were provided only in figures, the graphically presented data was converted to numerical data using a reliable and validated digital ruler software (WebPlotDigitizer) (Drevon et al., 2017; Rohatgi, 2015). We have previously confirmed the excellent inter‐rater reliability between two independent reviewers (intraclass correlation coefficient [2,1]: 0.999) (Iijima et al., 2022).

### 4.2 Determining differentially expressed genes in murine knee joint after traumatic injury

We have accessed archived RNA‐Seq data from young (3 months) and aged (15 months) mice from the study published by Sebastian et al. (2020), which yielded 2738 genes identified across the different time points (Day1, Week1, Week2, and Week6) after ACL rupture. We then isolated differentially expressed (false discovery rate <0.05) genes from young + ACL rupture at Week1 (935 genes), young + ACL rupture at Week2 (1121 genes), aged + ACL rupture at Week1 (1422 genes), and aged + ACL rupture at Week2 (1461 genes). Using VENN DIAGRAMS software (developed by Van de Peer Lab; http://bioinformatics.psb.ugent.be/webtools/Venn/), we finally identified 241 and 77 genes upregulated and downregulated uniquely in aged murine knee joint after ACL rupture, respectively (see Table S2). Further, we also identified 726 genes that were upregulated after ACL rupture in both young and aged murine knee joints. We then used these gene lists in the subsequent analyses for the tissue‐specific network construction shown below.

We have also repeated the same procedure for the different set of transcriptomic data of aged murine knee joint after DMM induction (Loeser, Olex, et al., 2012). We have accessed archived microarray data from young (3 months) and aged (12 months) mice from the study published by Loeser, Olex, et al. (2012), which yielded differentially expressed 549 probe sets with 432 genes in young or aged murine knee joint at 8 weeks after DMM induction. We then isolated 251 probe sets with 203 genes upregulated uniquely in aged murine knee joint at 8 weeks after DMM induction and used for subsequent analyses.

### 4.3 Determining genes associated with increased ECM stiffness of articular cartilage in murine knee joint

We sought to define genes associated with ECM stiffness in cartilage via a systematic literature search in accordance with the guidelines shown above (Higgins & Green, 2011; Moher et al., 2009; Shamseer et al., 2015; Stroup et al., 2000; Vesterinen et al., 2014). We included articles investigating the effects of loss‐ or gain‐of‐function approaches on ECM stiffness in cartilage of murine knee joint. No restrictions were set according to mouse age, strain, or article publication year. PubMed was used for electronic database search. Google Scholar was also used as a complementary search engine. A manual search of the reference lists of past reviews was performed (Bolia et al., 2022). Furthermore, citation searching was performed on the original records using Web of Science as recommended (Higgins & Green, 2011). The database search was performed on 15 November 2022. Electronic searches for PubMed used combined key terms, including “Animal, Genetically Modified” “Cartilage, Articular,” and “Osteoarthritis” using Medical Subject Headings terms.

A single reviewer (HI), as a content and statistical expert, assessed eligibility in accordance with the Cochran Handbook recommendations (Higgins & Green, 2011). The reviewer screened titles and abstracts yielded by the search. Full manuscripts of the articles that met the eligibility criteria were then obtained and reviewed. During these processes, the reviewer prepared and used simple, predesigned Google spreadsheets to assess eligibility by extracting study features.

When the loss‐of‐ or gain‐of‐function approach significantly (*p* < 0.05) changed ECM stiffness of articular cartilage evaluated by atomic force microscopy or similar assessment systems designed for assessing biophysical properties of cartilage, we classified the function of genes as (“+”) or (“−”), respectively; and as no effect (“=”) when no statistically significant difference was reported between the mice with the loss‐of‐ or gain‐of‐function approach and wild type control groups. More specifically, we categorized genes as (“+”) when (1) loss‐of‐function approach significantly decreased ECM stiffness or (2) gain‐of‐function approach significantly increased ECM stiffness.

### 4.4 Determining articular chondrocyte markers in murine knee joint

Articular chondrocyte markers were defined using previously published single‐cell RNA‐Seq data obtained from articular cartilage from 10‐week‐old adult male murine joint (*n* = 5) (Sebastian et al., 2021). After excluding immune and blood cells (CD45‐positive or TER119‐positive cells), the original study has identified 10 different clusters with distinct gene expression profiles, in which five clusters were annotated as “chondrocytes” (Sebastian et al., 2021). We defined chondrocyte markers as the genes which showed significantly higher enrichment to those five chondrocyte clusters compared to the other five clusters, resulting in 42 different genes (see Table S3).

### 4.5 Identification of cellular origin of cytokines using *Tabula Muris Senis*

We determined cell type and state identity by leveraging annotations provided in the *Tabula Muris Senis* following the “cell ontology” structure (Tabula Muris Consortium, 2020). The *Tabula Muris Senis* is a comprehensive resource for the cell biology community which offers a detailed molecular and cell‐type specific portrait of aging (Tabula Muris Consortium, 2020). We have used the data provided by droplet experiments. No restrictions were set according to mouse age, sex, and tissue type.

### 4.6 Construction of cytokine synergistic network regulating ECM homeostasis

Using systematic literature approach, we have manually curated pair of cytokines that display synergistic impact on ECM remodeling (i.e., MMPs and/or TIMPs) in articular cartilage. For the cytokine of interest, we have focused on cytokines that display synergistic effect with OSM and/or IL6. PubMed was used for electronic database search which was performed on 14 January 2023. Google Scholar was also used as a complementary search engine. Electronic searches for PubMed used combined key terms, including “Chondrocytes,” “Cartilage,” “Interleukin‐6,” “Oncostatin M,” and “Drug Synergism” using Medical Subject Headings terms. To ensure methodological rigor, the literature search was conducted in accordance with the PRISMA statement (Moher et al., 2009) and PRISMA‐P (Shamseer et al., 2015).

A single reviewer (HI), as a content and statistical expert, assessed eligibility. The reviewer screened titles and abstracts yielded by the search. Full manuscripts of the articles that met the eligibility criteria were then obtained and reviewed. During these processes, the reviewer prepared and used simple, predesigned Google spreadsheets to assess eligibility by extracting study features.

When the combination of two cytokines significantly (*p* < 0.05) changed ECM‐related genes or proteins in articular chondrocytes compared to single treatment alone, we classified the pair of cytokines as increased (“+”) or decreased (“−”), or no significant effect (“=”).

### 4.7 Pharmacological validation using microarray

We accessed the archived microarray data collected at 4 h after OSM or IL6 supplementation (100 ng/mL) to murine articular chondrocyte (Liu et al., 2015). The bioinformatics analysis conducted by the authors, Liu et al. (2015), revealed 2373 and 961 differentially (false discovery rate <0.05) expressed genes after OSM and IL6 treatments, respectively. We then used these gene lists in the subsequent analyses for the tissue‐specific network construction shown below. To prioritize genes which displayed drastic changes by OSM supplementation, we isolated nine genes significantly upregulated (mean expression >10, log fold change >2.5).

### 4.8 Defining a set of genes of “inflammation” phenotype of people with KOA

We have defined the genes associated with “inflammation” phenotype of people with KOA in accordance with single‐cell RNA‐Seq data from KOA cartilage samples (*n* = 131; mean age: 66.6 years old; 61.8%females; mean joint space narrowing score: 3.91) (Yuan et al., 2020). Using an unsupervised clustering approach, the cartilage samples were categorized into 1 (*n* = 81; 61.8%), 2 (*n* = 24; 18.3%), 3 (*n* = 10; 7.6%), and 4 (*n* = 16; 12.2%) according to the top 4000 most variable genes (Yuan et al., 2020). People classified into the inflammation phenotype (mean age: 71.0 years old; 56.3% females; median Kellgren and Lawrence grade 4) displayed more severe phenotype, as evidenced by severe joint space narrowing (mean joint space narrowing score: 4.38) (Yuan et al., 2020).

### 4.9 Meta‐analysis

To characterize age‐related alterations in articular cartilage and/or subchondral bone of murine knee joints to traumatic injury, we calculated pooled estimates and 95% confidence intervals for standardized mean differences (SMD) of outcome measures using the DerSimonian‐Laird method (Deeks & Higgins, 2010). This method considers the precision of individual studies and the variation between studies and weighs each study accordingly. SMD were calculated using the mean between‐group difference (young vs. young + injury, aged vs. aged + injury) divided by the pooled standard deviation (Deeks & Higgins, 2010). Meta‐analyses were performed using RevMan version 5.3 (Nordic Cochrane Centre, Cochrane Collaboration, Copenhagen, Denmark). Study heterogeneity, defined as the intertrial variation in study outcomes, was assessed using *I* ^2^, which is the proportion of total variance explained by intertrial heterogeneity (Higgins et al., 2003). To standardize semiquantitative scores of cartilage degeneration, all histological scores provided in each included study were converted to 0–100 and recalculated as in a previous meta‐analysis (van Middelkoop et al., 2016), with higher score indicates severe cartilage degeneration.

### 4.10 Construction of tissue‐specific network

Tissue‐specific networks were constructed using HumanBase web tool (Greene et al., 2015) with the genes of interest used an input. The data of tissue‐specific network data were based on several data types that constituted the underlying network, including experimentally produced protein–protein interactions (http://hb.flatironinstitute.org/data), and the interaction confidence is the edge weight assigned from the algorithm used to create this compendium network. As the tissue‐specific network was established based on human tissue, all the gene symbols were translated into human gene symbols prior to the analysis.

### 4.11 Functional characterization of transcriptome using GO enrichment analysis

To determine the biological function of genes of interests, GO enrichment analysis (biological process) was performed by Enrichr software (Kuleshov et al., 2016). REVIGO software (Supek et al., 2011) was applied to summarize redundant GO terms and visualize the summarized results.

### 4.12 GSEA

ssGSEA was performed using GSEA web tool provided by Broad Institute Website (https://www.gsea‐msigdb.org/gsea/index.jsp) with gene scores defined by log_2_ fold change of gene expression profiles of aged + ACL rupture at Week1 and 2. As a gene set, we have used genes associated with increased ECM stiffness in cartilage we have defined above.

### 4.13 Network propagation using RWR on cartilage‐specific global network

On cartilage‐specific global network, RWR was performed by R/Bioconductor package RandomWalkRestartMH (Valdeolivas et al., 2019) with the *Osmr* gene used as a seeded node. After iteratively reaching stability, the affinity score of all nodes in the given network to *Osmr* node were obtained. Cartilage‐specific global network was constructed using a gold standard data set (i.e., already known gene interactions) downloaded from HumanBase software (https://hb.flatironinstitute.org/download) (Greene et al., 2015). In the statistical analysis that characterize the relationship between the pseudo‐activated genes (affinity score >0) and interest of phenotype, we have excluded the 1284 genes that were significantly changed after traumatic injury in young mice to dissect the effects of age‐specific transcriptomic response (i.e., OSM‐OSMR axis) by removing the injury effects.

### 4.14 Unsupervised machine learning

PCA was performed for data reduction to identify the principal components that represent differences in the given data sets using JMP Pro 16 software (SAS Institute, Cary, NC). PCA produces linear combinations of the original variables to generate the principal components (Wold et al., 1987), and visualization was generated by projecting the original data to the first two principal components.

### 4.15 Cytokine inference

To define enriched cytokine signaling signatures of given gene expression data, cytokine inference was performed using CytoSig software (Jiang et al., 2021). CytoSig analyzes defined cytokine signatures that are differentially expressed when a cell is exposed to a specific cytokine (that is name giving for the respective cytokine signature). This study used the data of transcriptomic response (i.e., log_2_ fold change) of 58 genes related to ECM remodeling across the two groups (young + ACL rupture vs. aged + ACL rupture) over time was used as an input.

### 4.16 WGCNA

This study used the WGCNA package to build a weighted gene co‐expression network using the archived RNA‐Seq database (GSE114007) (Fisch et al., 2018), which finally yielded 13,729 genes identified across the different data sets after filtering low expression genes. We used filterByExpr function for count data in RNA‐Seq data (Chen et al., 2016). The key parameter, β, for weighted network construction was optimized to maintain both the scale‐free topology and sufficient node connectivity as recommended in the manual. A topological overlap matrix (TOM) was then formulated based on the adjacency values to calculate the corresponding dissimilarity (1‐TOM) values. Module identification was accomplished with the dynamic tree cut method by hierarchically clustering genes using 1‐TOM as the distance measure with a minimum size cutoff of 30 and a deep split value of 2 for the resulting dendrogram. A module preservation function was used to verify the stability of the identified modules by calculating module preservation and quality statistics in the WGCNA package (Langfelder & Horvath, 2008).

### 4.17 Drug repurposing via ConnectivityMap analysis

We explored chemical compounds that could possibly reverse OSM signaling, injured aged knees, and inflammation phenotype in KOA using ConnectivityMap (Subramanian et al., 2017). Using the online interface clue.io (https://clue.io/), we submitted the genes with OSM signaling pathway, age‐related stress response, and inflammation phenotype to calculate a “tau” connectivity score to gene expression signatures experimentally induced by various perturbations in nine cell lines. Gene list for OSM signaling pathway was obtained from “WikiPathway_2021_Human” via Enrichr (https://maayanlab.cloud/Enrichr/). As all the included genes across three conditions (i.e., OSM signaling, age‐related stress response, and inflammation phenotype) were upregulated genes, the subsequent analysis has focused on a negative tau score (gene expression signature of a perturbation opposes the submitted query). Recommended thresholds for further consideration of results are tau of below −90 (https://clue.io/connectopedia/connectivity_scores). Of 2837 compounds that were evaluated in clue.io, we shortlisted compounds where the summary tau across cell lines were lower than −90.

### 4.18 Statistical analysis

All statistical analyses were performed using JMP Pro 16 software (SAS Institute, Cary, NC) or SPSS Statistics for Windows, Version 28.0 (IBM Corp., NY, USA). Except where indicated, data are displayed as means, with uncertainty expressed as 95% confidence intervals (mean ± 95% CI). Two‐tailed Student's *t* test, linear regression analysis, logistic regression analysis, or Fisher's exact test were used for statistical analyses. We checked the features of the regression model by comparing the residuals versus fitted values (i.e., the residuals had to be normally distributed around zero) and independence between observations. No correction was applied for multiple comparison because outcomes were determined a priori and were highly correlated. No statistical analyses included confounders (e.g., body mass in each animal) due to the small sample size. We conducted a complete‐case analysis in the case of missing data. In all experiments, *p*‐values <0.05 were considered statistically significant. Throughout this text, “*n*” represents the number of independent observations of knees or cells from different animals. Specific data representation details and statistical procedures are also indicated in the figure legends.

## AUTHOR CONTRIBUTIONS

All authors made substantial contributions in the following areas: (1) conception and design of the study, acquisition of data, analysis and interpretation of data, drafting of the article; (2) final approval of the article version to be submitted; and (3) agreement to be personally accountable for the author's own contributions and to ensure that questions related to the accuracy are appropriately investigated, resolved, and the resolution documented in the literature. The specific contributions of the authors are as follows: Hirotaka Iijima, Fan Zhang, Yusuke Matsui provided the concept, idea and experimental design for the studies. Hirotaka Iijima, Fabrisia Ambrosio, Yusuke Matsui wrote the manuscript. Hirotaka Iijima, Fan Zhang, Fabrisia Ambrosio, Yusuke Matsui provided data collection, analyses, interpretation and review of the manuscript. Hirotaka Iijima obtained funding for the studies.

## CONFLICT OF INTEREST STATEMENT

The authors declare no competing interests.

## Supporting information


Figure S1–S9

Table S1–S5

## Data Availability

The raw data that support the experimental findings are included in article supplementary material. Any additional information required to reanalyze the data reported in this work is available from the corresponding author upon request.
