# Supplementary material for: Network‐based cytokine inference implicates Oncostatin M as a driver of an inflammation phenotype in knee osteoarthritis
Source: Aging Cell. 2023 Dec 18;23(2):e14043. doi: 10.1111/acel.14043 (PMC10861212; doi:10.1111/acel.14043)
Supplement: Supplementary file 1 — Figure S1–S9 [file ACEL-23-e14043-s002.docx]

**Supplementary Online Content**

*Network-based cytokine inference implicates Oncostatin M as a driver of an inflammation phenotype
in knee osteoarthritis*

*Hirotaka Iijima, PhD, PT^1,2,3,4,5^, Fan Zhang, PhD^6,7^, Fabrisia Ambrosio, PhD, MPT^1,2,3^, †Yusuke Matsui, PhD^5,8^

^1^Discovery Center for Musculoskeletal Recovery, Schoen Adams Research Institute at Spaulding, Charlestown, MA

^2^Department of Physical Medicine & Rehabilitation, Harvard Medical School, Boston, MA

^3^Department of Physical Medicine & Rehabilitation, Spaulding Rehabilitation Hospital, Charlestown, MA

^4^Institute for Advanced Research, Nagoya University, Nagoya, Japan

^5^Biomedical and Health Informatics Unit, Graduate School of Medicine, Nagoya University, Nagoya, Japan

^6^Department of Medicine Division of Rheumatology, University of Colorado School of Medicine, Aurora, CO

^7^Department of Biomedical Informatics Center for Health AI, University of Colorado School of Medicine, Aurora, CO

^8^Institute for Glyco-core Research, Tokai National Higher Education and Research System, Nagoya University, Nagoya, Japan

*Corresponding author. hiijima1@mgh.harvard.edu, †Co-corresponding author. matsui@met.nagoya-u.ac.jp

**The PDF file includes:**

Figure S1. Meta-analysis revealed that traumatic injury increased subchondral bone thickness in aged mice, but not in young mice

Figure S2. Traditional GO enrichment analysis did not identify ECM remodeling as a primary biological function of genes upregulated specific to aged mice after traumatic injury

Figure S3. Non-cartilage-specific network analysis did not identify ECM remodeling as a primary biological function of genes upregulated specific to aged mice after mechanical overloading

Figure S4. Cartilage-specific network analysis identified altered response to oxidate stress in downregulated genes of aged murine knee joint after traumatic injury

Figure S5. Cartilage-specific network analysis identified ECM remodeling module in upregulated and downregulated genes of aged murine knee joint after traumatic injury

Figure S6. Cartilage-specific network analysis identified ECM remodeling module in transcripts of aged murine knee joint at 8 weeks after DMM induction

Figure S7. ECM remodeling was a common transcriptomic signature of traumatic injury

Figure S8. Age-related aberrant ECM remodeling genes were associated with chondrocyte markers

Figure S9. Integrated analysis of RNA-seq revealed unique contribution of OSM on age-related stress response in murine chondrocytes

Supplemental references

This supplementary material has been provided by the authors to give readers additional information.

**
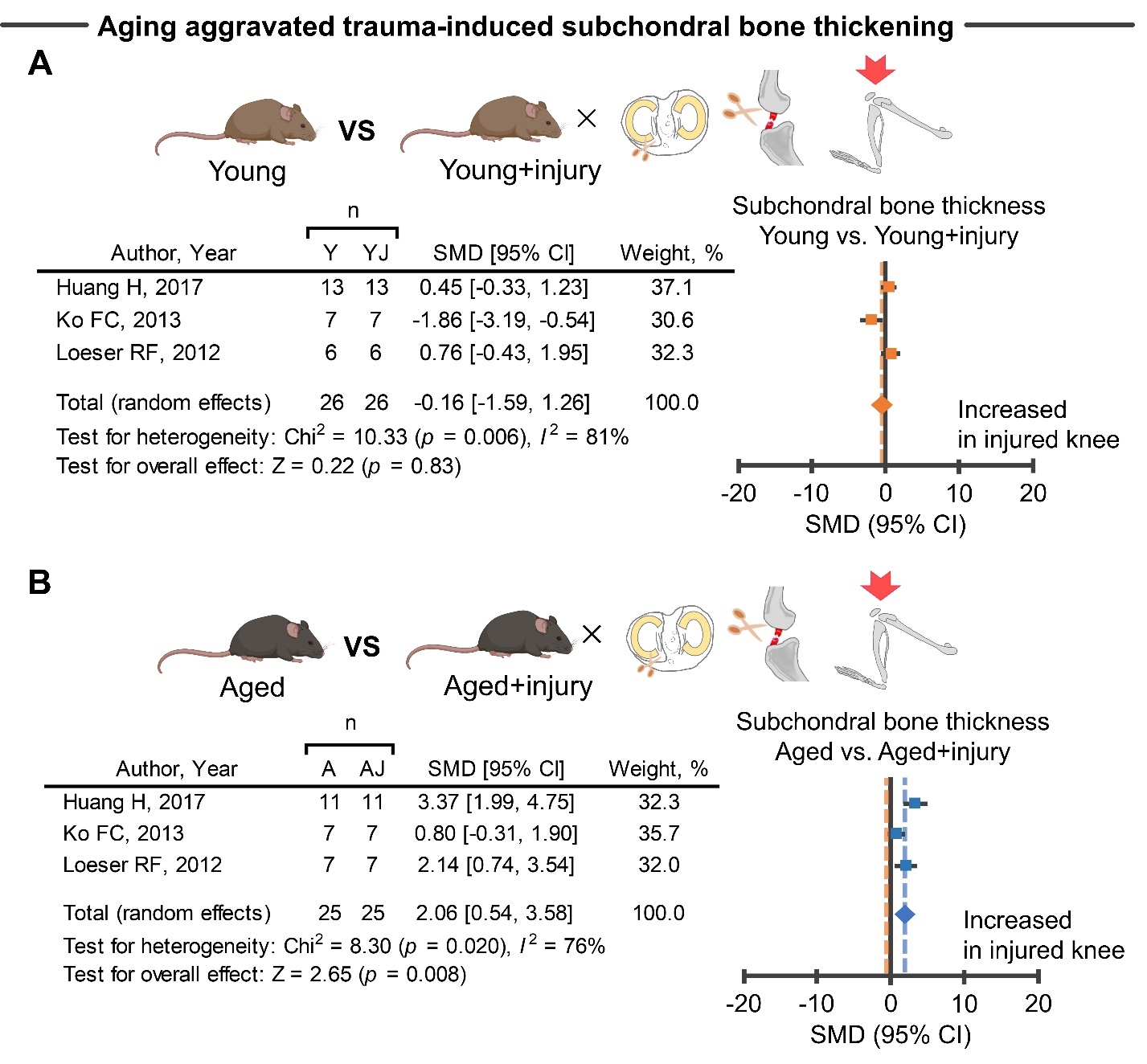
**

**Figure S1. Meta-analysis revealed that traumatic injury increased subchondral bone thickness in aged mice, but not in young mice**

Meta-analysis from three studies summarizing the influence of traumatic injury on subchondral bone thickness in young (**A**) and aged (**B**) mouse knee joints. The forest plot displays relative weight of the individual studies, SMDs, and 95% CIs. Diamonds indicate the global estimate and its 95% CI. The orange dotted line in the lower panel indicates the average SMD of young mice. Portions of the figures were created with biorender.com.

*Abbreviation: 95% CI: 95% confidence interval; A, aged; AJ, aged+injury; Y, young; YJ, young+injury; SMD*, *standardized mean difference*.


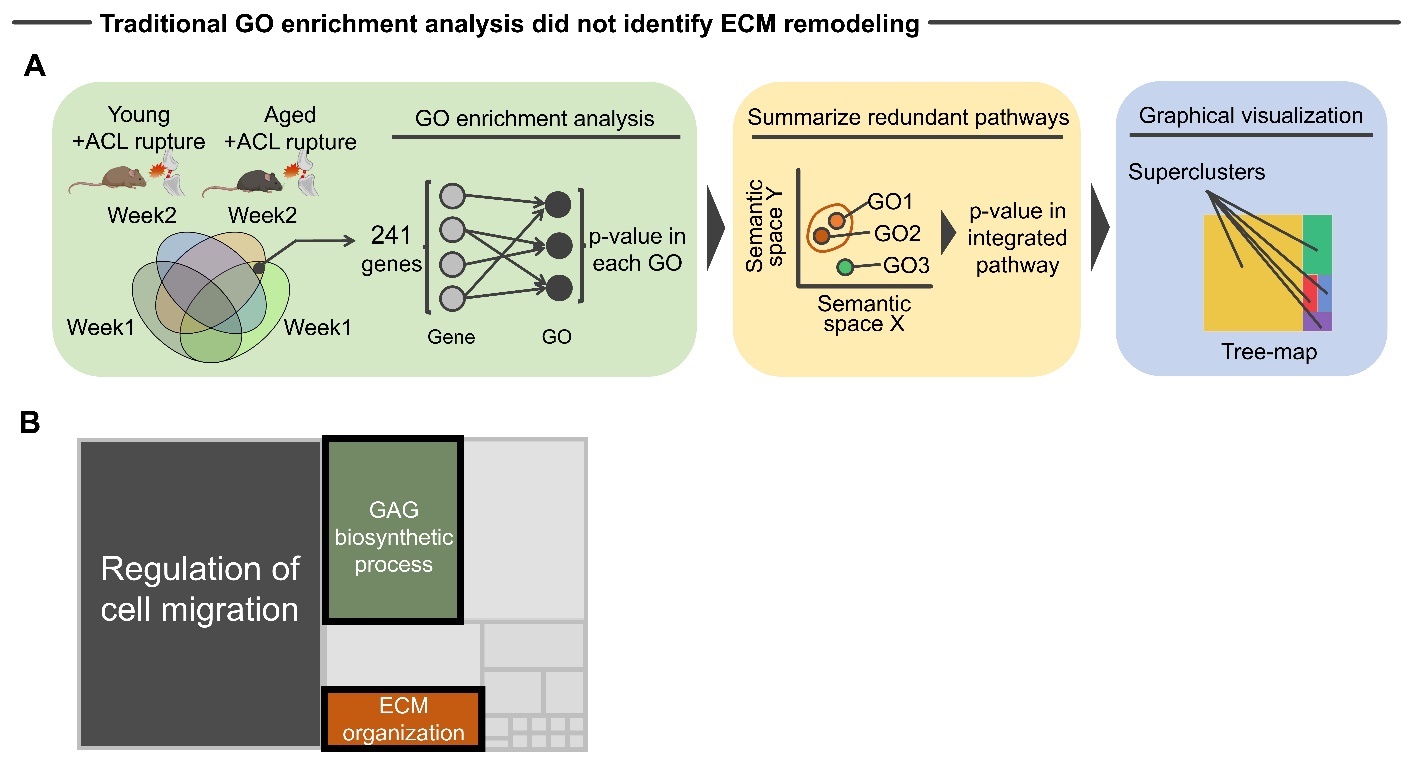


**Figure S2. Traditional GO enrichment analysis did not identify ECM remodeling as a primary biological function of genes upregulated specific to aged mice after traumatic injury**

**A,** Schematic showing the analytical flow of GO enrichment analysis with subsequent summarizing redundant GO of 241 age-related stress response genes. REVIGO was used to summarize redundant pathways. **B,** GO enrichment tree-map of biological processes for the 241 genes upregulated specific to aged mice after ACL rupture. Each rectangle represents a supercluster GO, visualized with different colors. The sizes of the rectangles were adjusted to reflect the p-value of the GO term calculated by Top GO (i.e., the larger the rectangle, the more significant the GO-term). Portions of the figures were created with biorender.com.

*Abbreviation: ACL, anterior cruciate ligament; ECM, extracellular matrix; GAG, glycosaminoglycan; GO, gene ontology.*


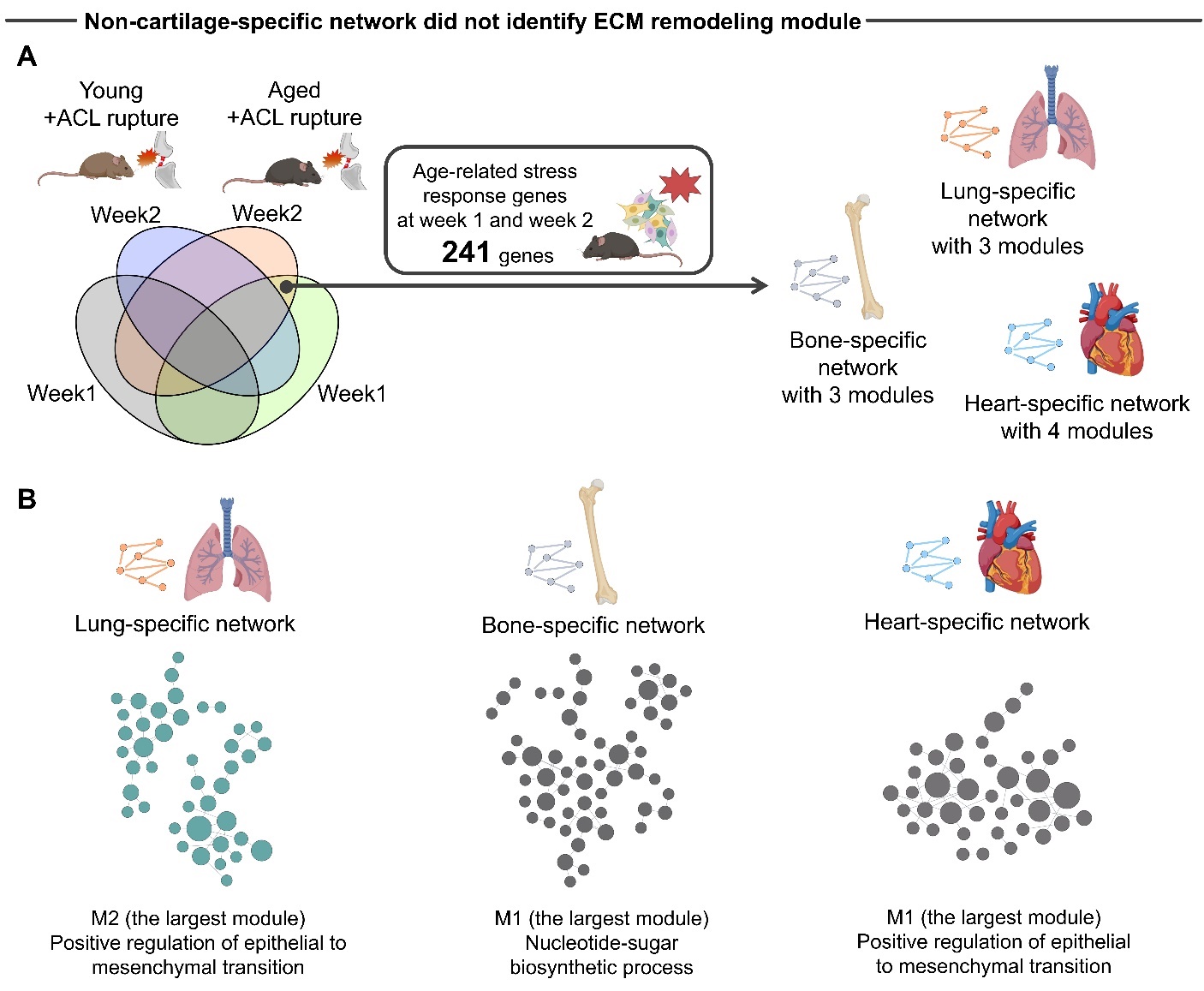


**Figure S3.** **Non-cartilage-specific network analysis did not identify ECM remodeling as a primary biological function of genes upregulated specific to aged mice after traumatic injury**

**A,** Schematic showing the analytical flow of non-cartilage-specific network construction (i.e., lung, bone, and heart) using 241 age-related stress response genes. **B,** The largest module identified by lung-, bone-, and heart-specific network construction. In the network, each node and edge represent gene and gene-gene interaction, respectively. Portions of the figures were created with biorender.com.

*Abbreviation: ACL, anterior cruciate ligament; ECM, extracellular matrix.*


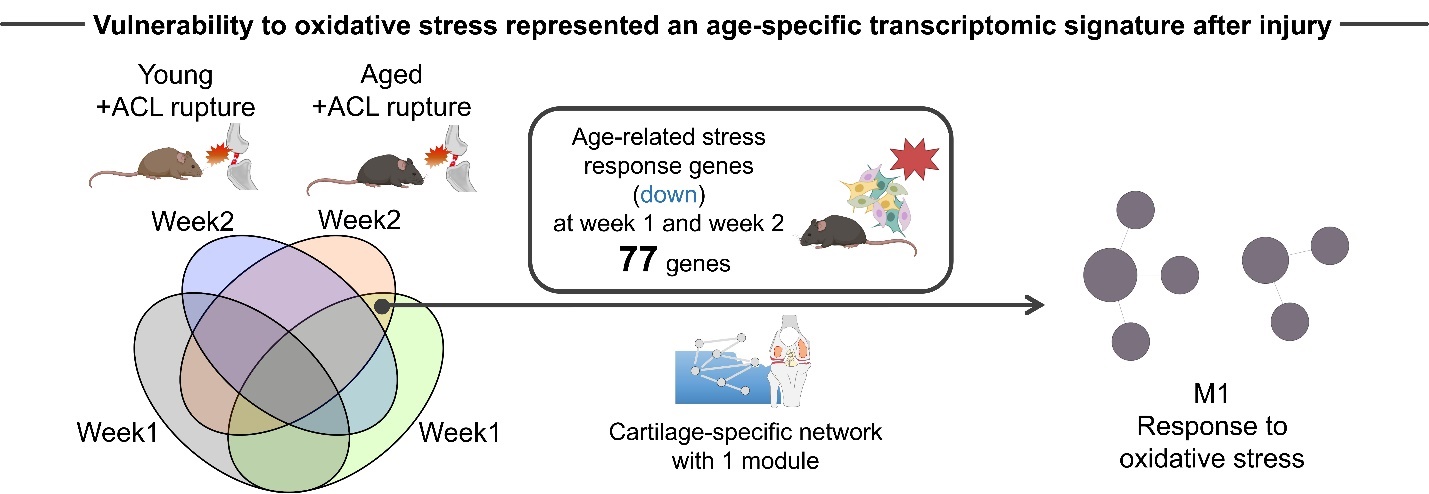


**Figure S4. Cartilage-specific network analysis identified altered response to oxidate stress in downregulated genes of aged murine knee joint after traumatic injury**

We identified 77 age-related stress response genes that were significantly “downregulated” in aged+ACL rupture (versus uninjured contralateral knee) across the different time point (week1 and week2) but not in young+ACL rupture. From 77 genes, a cartilage-specific network was constructed using HumanBase software(Greene et al., 2015), which identified only one module associated with response to oxidative stress. Portions of the figures were created with biorender.com.

*Abbreviation: ACL, anterior cruciate ligament.*

**
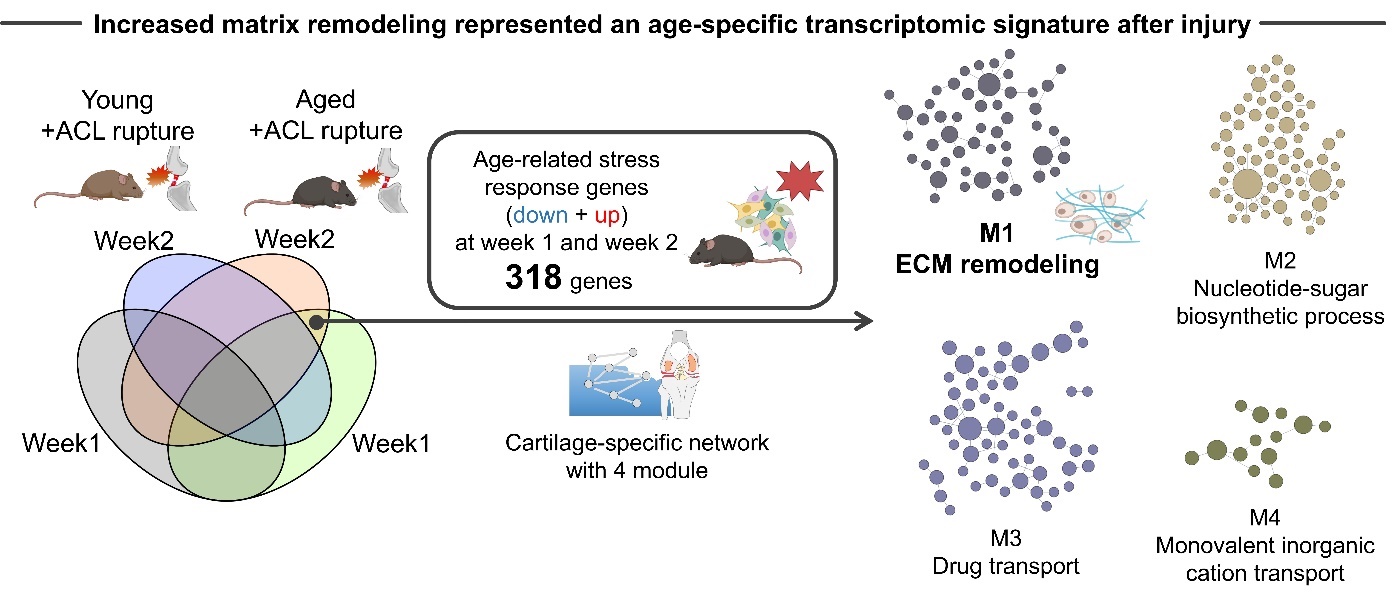
**

**Figure S5. Cartilage-specific network analysis identified ECM remodeling module in upregulated and downregulated genes of aged murine knee joint after traumatic injury**

We identified 318 age-related stress response genes that were significantly “upregulated” or “downregulated” in aged+ACL rupture (versus uninjured contralateral knee) across the different time point (week1 and week2) but not in young+ACL rupture. From 318 genes, a cartilage-specific network was constructed using HumanBase software(Greene et al., 2015), which still identified a large module annotated to ECM remodeling. Portions of the figures were created with biorender.com.

*Abbreviation: ACL, anterior cruciate ligament; ECM, extracellular matrix*.


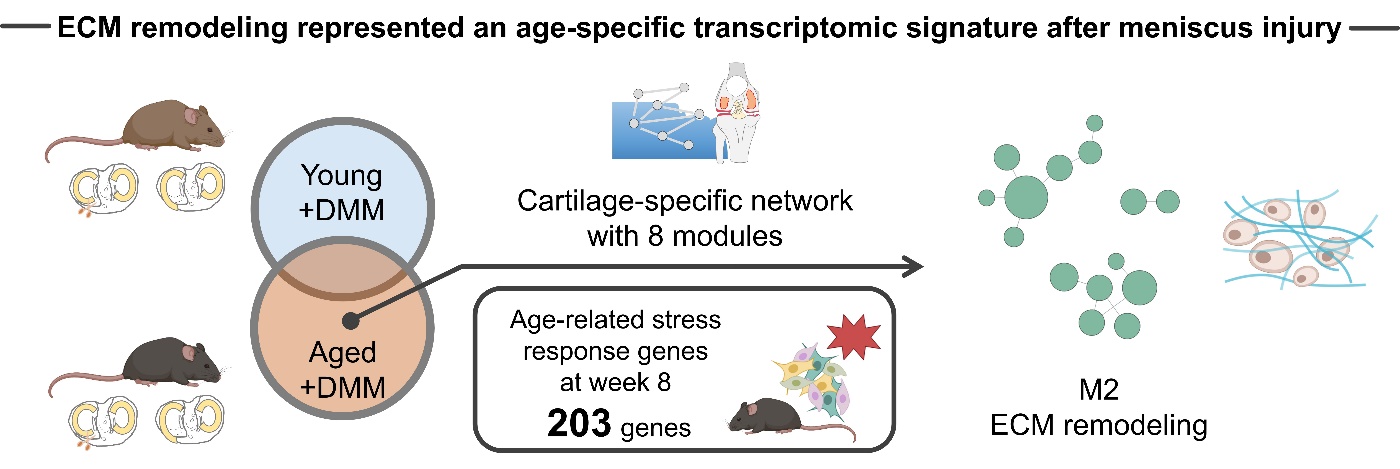


**Figure S6. Cartilage-specific network analysis identified ECM remodeling module in transcripts of aged murine knee joint at 8 weeks after DMM induction**

We identified 203 genes that were significantly upregulated in aged+DMM, but not in young+DMM, at 8 weeks after surgery(Loeser et al., 2012). From 203 genes, a cartilage-specific network was constructed using HumanBase software(Greene et al., 2015), which identified module associated with ECM remodeling. Portions of the figures were created with biorender.com.

*Abbreviation: DMM, destabilized medial meniscus; ECM, extracellular matrix*.


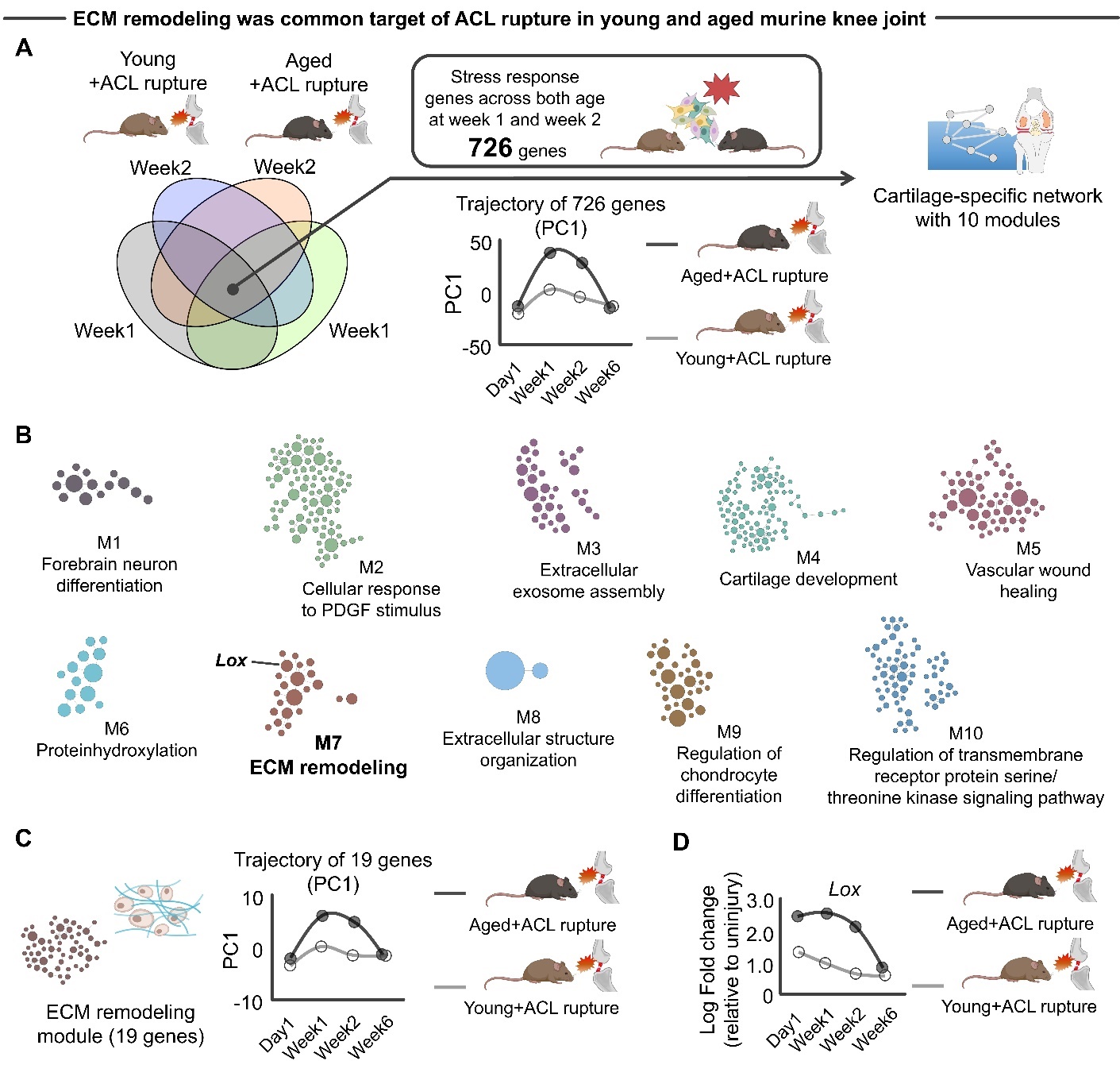


**Figure S7. ECM remodeling was a common transcriptomic signature of traumatic injury**

**A,** From archived RNA-seq data with 2,738 genes(Sebastian et al., 2020), we identified 726 genes that were significantly upregulated in both young and aged knee joints after ACL rupture across the different time points (week1 and week2). PCA revealed PC1 with 726 genes displayed different trajectory between young and aged mice after ACL rupture with the largest difference at week1 and week2. From 726 genes, a cartilage-specific network was constructed using HumanBase software(Greene et al., 2015). **B,** The cartilage-specific network included 10 modules with include “ECM remodeling”, in which a major enzyme involved in collagen crosslinking, *Lox*, was one of the hub genes. **C,** PCA revealed that PC1 of ECM remodeling module genes (19 genes) displayed different trajectory between young and aged mice after ACL rupture with the largest difference at week1 and week2. **D,** Transcriptomic response of *Lox* after ACL rupture was greater in aged murine knee joint compared to young counterparts. Portions of the figures were created with biorender.com.

*Abbreviation: ACL,* *anterior cruciate ligament; ECM, extracellular matrix; PC, principal component; PCA, principal component analysis.*


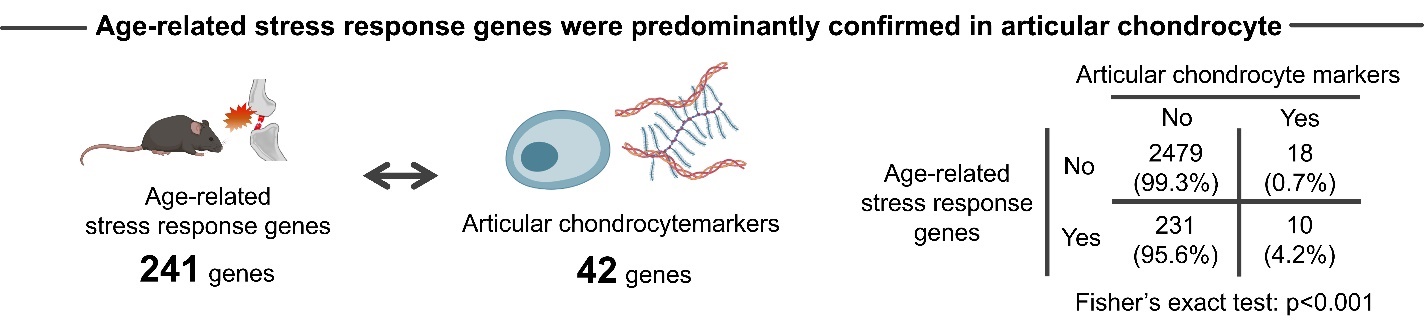


**Figure S8. Age-related aberrant ECM remodeling genes were associated with chondrocyte markers**

**A,** Association between 241 genes upregulated in aged+ACL rupture and articular chondrocyte markers. We defined 42 articular chondrocyte markers from single cell RNA-seq data from murine knee joints(Sebastian et al., 2021). Values in 2×2 contingency indicate number of genes (percentage), in which statistical analysis was performed using Fisher’s exact test (p <0.001). Portions of the figures were created with biorender.com.

*Abbreviation: ACL, anterior cruciate ligament.*

**
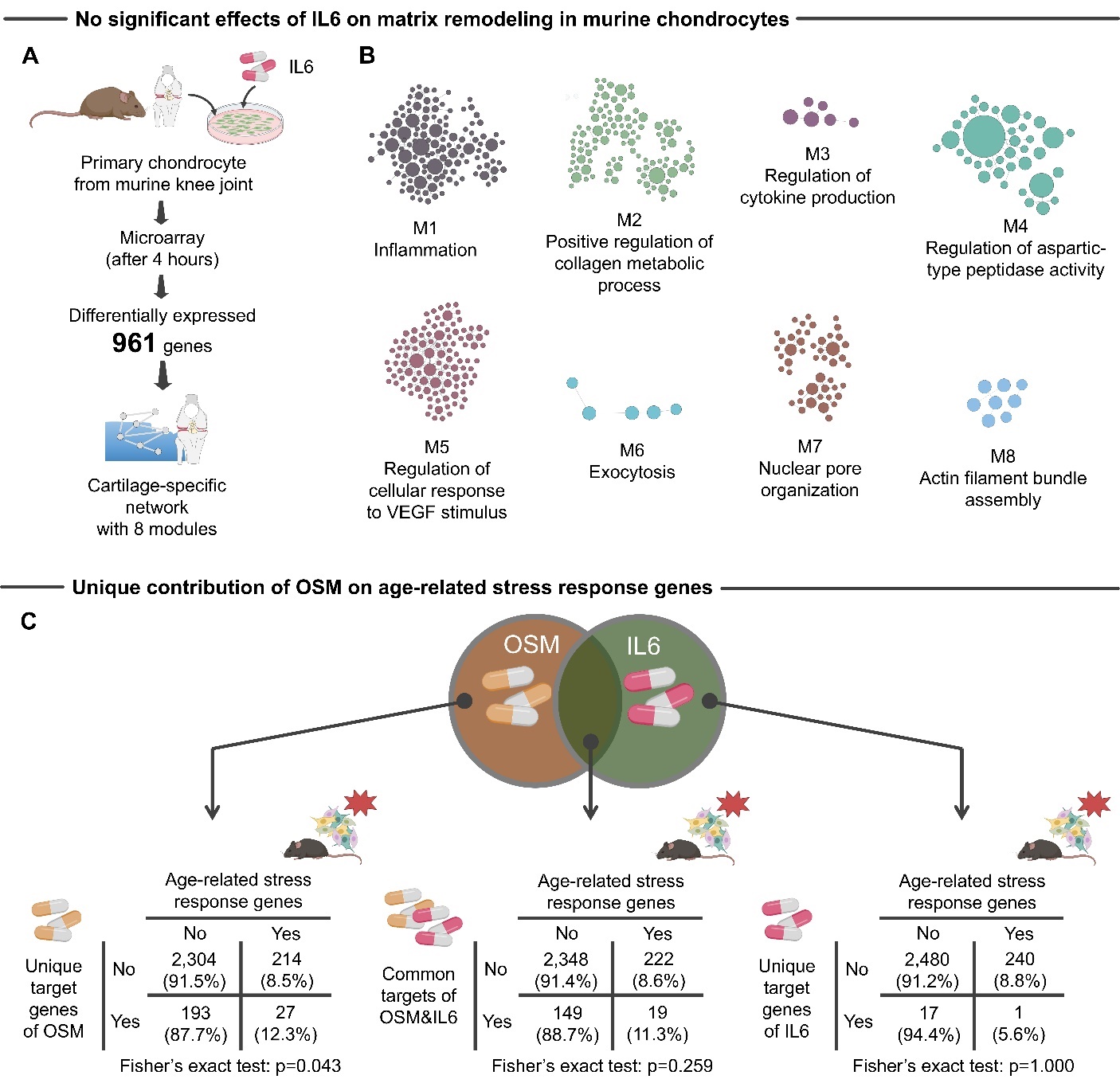
**

**Figure S9. Integrated analysis of RNA-seq revealed unique contribution of OSM on age-related stress response in murine chondrocytes**

**A,** Schematic showing the experimental flow of microarray data +/- IL6 supplementation to primary murine chondrocytes(Liu et al., 2015). The microarray revealed 961 differentially expressed gene at 4 hours after IL6 supplementation (100ng/mL) to chondrocytes. **B,** Cartilage-specific network constructed based on 961 differentially expressed genes, identifying no module associated with ECM remodeling. **C,** Association of differentially expressed genes unique to OSM, IL6, and their common targets with age-related stress response genes. Values in the 2×2 contingency table indicate the number of genes (percentage). Statistical analyses were performed using Fisher’s exact test (**C**). Portions of the figures were created with Biorender.com.

*Abbreviation: ECM, extracellular matrix.*

**Supplemental reference**

Greene, C. S., Krishnan, A., Wong, A. K., Ricciotti, E., Zelaya, R. A., Himmelstein, D. S., . . . Troyanskaya, O. G. (2015). Understanding multicellular function and disease with human tissue-specific networks. *Nat Genet, 47*(6), 569-576. doi:10.1038/ng.3259

Liu, X., Liu, R., Croker, B. A., Lawlor, K. E., Smyth, G. K., & Wicks, I. P. (2015). Distinctive pro-inflammatory gene signatures induced in articular chondrocytes by oncostatin M and IL-6 are regulated by Suppressor of Cytokine Signaling-3. *Osteoarthritis Cartilage, 23*(10), 1743-1754. doi:10.1016/j.joca.2015.05.011

Loeser, R. F., Olex, A. L., McNulty, M. A., Carlson, C. S., Callahan, M. F., Ferguson, C. M., . . . Fetrow, J. S. (2012). Microarray analysis reveals age-related differences in gene expression during the development of osteoarthritis in mice. *Arthritis Rheum, 64*(3), 705-717. doi:10.1002/art.33388

Sebastian, A., McCool, J. L., Hum, N. R., Murugesh, D. K., Wilson, S. P., Christiansen, B. A., & Loots, G. G. (2021). Single-Cell RNA-Seq Reveals Transcriptomic Heterogeneity and Post-Traumatic Osteoarthritis-Associated Early Molecular Changes in Mouse Articular Chondrocytes. *Cells, 10*(6). doi:10.3390/cells10061462

Sebastian, A., Murugesh, D. K., Mendez, M. E., Hum, N. R., Rios-Arce, N. D., McCool, J. L., . . . Loots, G. G. (2020). Global Gene Expression Analysis Identifies Age-Related Differences in Knee Joint Transcriptome during the Development of Post-Traumatic Osteoarthritis in Mice. *Int J Mol Sci, 21*(1). doi:10.3390/ijms21010364
